# Identification of Proteins Related to Epigenetic Regulation in the Malignant Transformation of Aberrant Karyotypic Human Embryonic Stem Cells by Quantitative Proteomics

**DOI:** 10.1371/journal.pone.0085823

**Published:** 2014-01-17

**Authors:** Yi Sun, Yixuan Yang, Sicong Zeng, Yueqiu Tan, Guangxiu Lu, Ge Lin

**Affiliations:** 1 Institute of Reproductive and Stem Cell Engineering, Central South University, Changsha, China; 2 National Engineering and Research Center of Human Stem Cells, Changsha, China; 3 Key Laboratory of Stem Cells and Reproductive Engineering, Ministry of Health, Changsha, China; 4 Key Laboratory of Molecular Biology for Infectious Diseases of Ministry of Education of China, The Second Affiliated Hospital, Chongqing Medical University, Chongqing, China; University of Central Florida, United States of America

## Abstract

Previous reports have demonstrated that human embryonic stem cells (hESCs) tend to develop genomic alterations and progress to a malignant state during long-term in vitro culture. This raises concerns of the clinical safety in using cultured hESCs. However, transformed hESCs might serve as an excellent model to determine the process of embryonic stem cell transition. In this study, ITRAQ-based tandem mass spectrometry was used to quantify normal and aberrant karyotypic hESCs proteins from simple to more complex karyotypic abnormalities. We identified and quantified 2583 proteins, and found that the expression levels of 316 proteins that represented at least 23 functional molecular groups were significantly different in both normal and abnormal hESCs. Dysregulated protein expression in epigenetic regulation was further verified in six pairs of hESC lines in early and late passage. In summary, this study is the first large-scale quantitative proteomic analysis of the malignant transformation of aberrant karyotypic hESCs. The data generated should serve as a useful reference of stem cell-derived tumor progression. Increased expression of both HDAC2 and CTNNB1 are detected as early as the pre-neoplastic stage, and might serve as prognostic markers in the malignant transformation of hESCs.

## Introduction

Human embryonic stem cells (hESCs) derived from the inner cell mass of human embryos have held great promise for future cell- and tissue-replacement therapy because of their unique capacity to self-renew and to differentiate into any cell type. However, concerns have been raised with regard to the safety of hESCs, which commonly undergo adaptive changes during prolonged passaging *in vitro*, such as increased growth rate, reduced apoptosis and especially karyotypic changes [1]–[8]. With these changes, the culture adaptation of hESCs tends towards a transformed phenotype of tumor stem cells and emphasizes the need for thorough analysis of cells destined for clinical applications [2], [3], [5], [6], [8], [9]. It is important to realize that hESCs preparations destined for clinical use are free from cancer-associated genomic alterations and the ability to identify karyotypically abnormal hESCs in contrast to normal hESC can provide further insights into this abnormality and provide a screening approach to detect abnormal cells. Thus, the discovery of cell proteins that improve characterization, and are capable of distinguishing particular hESCs populations has a high potential for providing an invaluable resource for culturing hESC lines in clinical practice.

By contrast, many studies have indicated that genetic changes of transformed hESCs are associated with neoplasia, which can affect apoptotic pathways, differentiation control or cell cycle, and which may arise in precancerous cells, resulting in uncontrolled and increased growth [2], [3], [5], [6], [8], [9]. Although several mechanisms may contribute to the genomic instability of hESCs, such as abnormal DNA repair systems [10], hypoxia [11] and long interspersed element 1 s retrotransposition accommodation [12], the molecular mechanisms underlying the progression and metastasis of transformed hESCs remain poorly understood. Thus, transformed hESCs may serve as an excellent model for characterizing the initial stages that determine transition of embryonic stem cells into cancerous stem cells.

To date, many attempts have been made to study the global genome of hESC and its chromatin state [5], [13]–[15]. However, characterization of the proteome of hESCs, especially of aberrant karyotypic hESCs, has just begun. Some of the proteomic studies have been performed on human ESCs and ECCs [16]–[19] including non-quantitative and quantitative analyses. However, although useful in providing critical information with regard the regulators of these two closely related, but developmentally distinct stem cells, proteomic studies do not permit differential analyses among these populations nor do they give quantitatively differential analyses of transformed hESCs at different stages of development.

Our previous reports indicated that during long-term culture, *ch*HES-3 cells [20], which were established in our laboratory, acquired genomic alterations. In addition, the anti-apoptotic and proliferative ability of transformed *ch*HES-3 gradually increased with increasing karyotypic complexity, which had a tendency to progress to malignant cells [8], [9]. Here, we employed ITRAQ-based quantitative and comparative proteomics [21]–[24] to quantify proteins of normal and aberrant karyotypic hESCs from simple to more complex karyotype abnormalities. We believe that such analyses will provide functional characterization to distinguish transformed and normal hESCs cells. Such information should advance understanding of the molecular mechanisms that regulate carcinogenesis of karyotypically abnormal hESCs. Additionally, such analyses could help identify the factors involved in hESCs proliferation, self-renewal and pluripotency, and thus contribute to the discovery of transformed phenotypic biomarkers for further monitoring the clinical safety and use of hESCs in therapeutic applications.

## Materials and Methods

### Cell Culture

The derivation experiment of hESCs was approved and guided by the local ethical committee of Reproductive and Genetic Hospital of CITIC-Xiangya, China. The hESC lines (*ch*HES-3) were established in our laboratory as previously described [20]. *ch*HES-3 was cultured in serum-free medium and on embryonic fibroblast cells(MEF), which were isolated from Chinese Kun-Min White mice at 12.5 d with a high density of 6–7×10^4^ cells/cm^2^ and were inactivated by mitomycin-C (10 µg/ml) [9]. Every 6–7 days, the *ch*HES-3 cells were passaged using mechanical dissection or a combination of 200 U/ml collagenase IV digestion reagent (Gibco-BRL, USA) followed by mechanical slicing. After multiple passaging, *ch*HES-3 cells acquired chromosomal abnormalities when cultured in this suboptimal culture conditions. By periodically examining karyotype of hESCs every 5–10 passages, we found that the karyotype of the *ch*HES-3 cell line displayed a slowly progressive changes. But there existed only one karyotype after 142 passages by karyotype analysis of standard G-banding [8], [9].

In this study, the *ch*HES-3 cells with normal (Normal), and karyotypically aberrant *ch*HES-3 cells that displayed a simple duplication karyotype (SIMP) and a complex karyotype (COMP) were grown on ICR MEF (Harlan, Indianapolis, IN, USA) with a low density of 2×10^4^ cells/cm^2^. Feeder cells were inactivated by treating with mitomycin C (Sigma-Aldrich, St. Louis, MO, USA) and cultured in serum-free DFSR medium, containing knock-out DMEM/F12 medium (Gibco-BRL, USA) that was supplemented with 15% serum replacement (Gibco-BRL, USA), 0.1 mM β-mercaptoethanol (Sigma, USA), 1% nonessential amino acids (Gibco-BRL, USA), 2 mM L-glutamine (Gibco-BRL, USA), and 4 ng/ml human recombinant basic fibroblast growth factor (Gibco-BRL, USA). The cells were passaged by mechanical disruption every 6 d.

The hESC lines were cultured under identical conditions over a 4 wk period to prevent culture variation, and were constantly monitored for any differentiation events by immunocytochemistry. To completely remove traces of feeder cells, hESCs were cultured on MEFs and subsequently passaged on matrigel-coated plates for five sub-cultures in conditioned media. Media that was prepared for feeder-free cultures was conditioned by exposure to MEFs for 24 h, which was then supplemented with an additional 4 ng/mL of bFGF. Conditioned media was filtered using a 0.2-mM filter. Before lysis, ESCs were washed three times with cold PBS to remove traces of cell-culture contaminants from the culture medium. The human embryonal carcinoma cell line (hECCs) NTERA-2 cl.D1 (EC) was obtained from the American Type Culture Collection (Manassas, Virginia, USA) and cultured on matrigel-coated plates under conditions described previously for this cell line [25]. All other hESC lines were established and cultured in our laboratory as previously reported [26].

### Karyotypic Analysis

Karyotype analysis by standard G-banding was performed with approximately 50 metaphases and three successive passages of hESCs were analyzed. Human ESCs cells were cultured in hESC medium containing 0.06 ug/ml colcemid (Sigma, USA) for 2.5 h. After washing with PBS for three times, the cells were incubated in hESC medium containing 0.05% trypsin and 0.53 mM EDTA (Gibco-BRL, USA), at 37°C for 5–10 min and harvested using standard procedures, followed by standard G-banding for karyotyping. At least 50 metaphase spreads were examined for each sample using an Olympus epi-fluorescence microscope BX51 (Olympus, Tokyo, Japan) with LUCIA KARYOTYPE software (Lucia, Praha, Czech Republic).

### Detection of hESC-specific Markers

Antibodies used for immunocytochemical staining and Western blot analyses are summarized in the Table S1. HESCs-specific surface markers consisting of OCT4, TRA-1-60, TRA-1-81, SSEA-4, SSEA-3 and SSEA1 were tested by immunostaining. Cells were fixed in 4% paraformaldehyde for 20 min, permeabilized with 0.2% Triton X-100 for 10 min, and blocked in 4% goat serum in PBS for 30 min. Cells were incubated with primary antibody overnight at 4°C. Next, the cells were stained with Alexa Fluor (Invitrogen, USA) secondary antibody for 1 h. Nuclei were then counterstained with 4′, 6-diamidino-2-phenylindole (DAPI, KPL, Gaithersburg, MD, USA). Alkaline phosphatase activity was detected according to the protocol of the Fast Red Substrate Pack (Invitrogen, USA).

### Protein Preparation and Quantification

Normal *ch*HES-3 cells (Normal) of passage number 30 (P30, P represents the hESCs culture passage in vitro), and karyotypically aberrant *ch*HES-3 cells with a simple duplication karyotype (SIMP) of passage number 72 (P72) and complex karyotype (COMP) of passage number 182 (P182) were cultured in 6-well tissue culture plates in clone-like clumps, wherein we selected out undifferentiated ES cells under an inverted microscope. HECCs were also collected in serum-free media. The isolated cells were washed three times with ice-cold PBS, and collected by centrifugation at 1000 rpm. Cells were suspended in 200 µl of cell lysis buffer (7 M urea, 1 mg/mL DNase I, 1 mM Na3VO4, and 1 mM PMSF) at 4°C. The cell lysate was subjected to intermittent sonication using a Vibra Cell™ high intensity ultrasonic processor (Jencon, Leighton Buzzard, Bedfordshire, UK). The remaining unbroken cells and debris were removed by centrifugation at 12, 000×g at 4°C for 10 min. Protein concentrations of the cleared lysates were determined by 2-D quantification kit (Amersham Biosciences, Uppsala, Sweden) according to the manufacturer’s instructions.

### Protein Digestion and iTRAQ Labeling

The approximately 100 µg of proteins were reduced with 5 mM tris-carboxyethyl phosphine hydrochloride (TCEP) for 60 min at 37°C, alkylated with 10 mM methylethanethiosulfonate (MMTS) for 20 min at room temperature (RT), and then diluted 10 times with deionized water prior to digestion with 20 µL of 0.25 µg/µL sequencing grade trypsin (Promega, WI, USA) overnight at 37°C. Samples were then air-dried using a Speedvac (Thermo Electron). Peptides generated this way were labeled with iTRAQ reagents according to the manufacturer’s supplied protocol (Applied Biosystems, MA, USA). Briefly, digested proteins were reconstituted in 30 µl of dissociation buffer (0.5 M TEAB) and mixed with 70 µl of ethanol-suspended iTRAQ reagents (one iTRAQ reporter tag per protein sample). The samples were labeled with the respective tags as follows: *ch*HES-3 cells subsets including Normal, SIMP and COMP *ch*HES-3 cells, were labeled with the reporter tags 114, 115, and 116 respectively, and hECCs cells were labeled with reporter tag 117. Labeling reactions were carried out at RT for 60 min before all samples were mixed in a single tube and air-dried using a Speedvac.

### Fractionation of Peptides by Isoelectric Focusing (IEF) on an Immobilized pH Gradient

iTRAQ-labeled tryptic peptide samples were dissolved in 300 µL of 8 M urea and 1% Pharmalyte (Amersham Biosciences). Samples were used to rehydrate IPG strips (pH 3−10, 18 cm long, Amersham Biosciences) for 14 h at 30 V. Peptides were subsequently focused successively for 1 h at 500 V, 1 h at 1000 V, 1 h at 3000 V and 8.5 h at 8000 V to give a total of 68 kV·h on IPGphor (Amersham Biosciences). The strips were then removed and quickly cut into 36×0.5 cm pieces. We performed peptide extractions by incubating the gel pieces in 100 µL of 2% acetonitrile and 0.1% formic acid for 1 h. These fractions were lyophilized in a vacuum concentrator and subjected to C-18 clean-up using a C18 Discovery DSC-18 SPE column (100 mg capacity, Supelco, Sigma-Aldrich). The cleaned fractions were then lyophilized again and stored at −20°C prior to mass spectrometric analysis.

### Mass Spectrometric Analysis Using Q-STAR and Data Analysis

Each cleaned-up peptide fraction was resuspended in 20 µl of Buffer A (0.1% formic acid in 2% acetonitrile). Ten microliters of sample was injected into the nano-LC−ESI−MS/MS system for each analysis. Mass spectrometry was performed using a QStar Elite Hybrid ESI Quadrupole time-of-flight tandem mass spectrometer (ESI-Q-TOF-MS/MS, Applied Biosystems, Framingham, MA, USA; MDS-Sciex, Concord, Ontario, Canada) coupled to an online capillary liquid chromatography system (Dionex Ultimate 3000, Amsterdam, The Netherlands). The peptide mixture was separated on a PepMap C-18 RP capillary column (Dionex) at 0.3 µl/min. A 125-min gradient was used, in which the gradient was started with 4% Buffer B (0.1% formic acid in 98% acetonitrile) and 96% Buffer A for 3 min, followed by 3 ramping gradients of 4−10% Buffer B for 7 min, 10−35% Buffer B for 55 min and 35−100% Buffer B for 25 min. This was then held in 100% Buffer B for 15 min and finally in 96% Buffer A for 20 min.

The mass spectrometer was set to perform data acquisition in the positive ion mode, with a selected mass range of 300−1800 m/z. The time of summation of MS/MS events was set to be 2s. This refers to the amount of time allowed for the machine to accumulate MS/MS events before switching back to the MS scan. The two most abundant charged peptides above a 20-count threshold were selected for MS/MS and dynamically excluded for 30 s with ±50 mDa mass tolerance. Protein identification and quantification for iTRAQ samples were carried out using ProteinPilot software (version 2.0; Applied Biosystems, MDS-Sciex). Following independent analyses, three datasets from biological replicates were searched as one. The search was performed against the International Protein Index (IPI) human database (version 3.41, date of release: March 2008, 72, 155 sequences). We searched databases by setting cysteine modification by MMTS as a fixed modification. Other parameters included mass tolerance of up to 0.2 Da, maximum of one missed cleavage of trypsin, oxidation of methionine, N-terminal iTRAQ labeling and iTRAQ labeled-lysine. Relative quantification of proteins in the case of iTRAQ was performed on the MS/MS scans and was the ratio of the areas under the peaks at 114, 115, 116 and 117 Da, which represented the masses of the tags that corresponded to the iTRAQ reagents used to label the samples. Statistical calculation for iTRAQ-based detection and relative quantification was performed using the Paragon Algorithm19 embedded within the ProteinPilot software.

Following data analysis by the ProteinPilot software, the protein summary results were exported into an Excel spreadsheet and manually inspected and processed. Briefly, for protein identification and quantitative analysis, 95% confidence intervals were used. Protein identification must be based on at least two unique peptides and the p-values for the relative quantification by iTRAQ must be *P*<0.05. Protein hits that did not satisfy these criteria were removed.

### PANTHER Analysis

The PANTHER database was used to elucidate the molecular function, biological process and signaling pathways associated with each individual protein (http://panther.appliedbiosystems.com/).

### Reanalysis of Microarray Data

Microarray data of normal *ch*HES-3 cells and aberrant *ch*HES-3 cells with a simple duplication karyotype (SIMP) and complex karyotype (COMP) were obtained from PubMed GEO datasets (GSM172579, GSM172580, GSM172581, GSM172582). To compare transcriptomic and proteomic data, all proteins with a SWISS-PROT accession number were selected, and these accessions were used to identify the relevant probeset identifiers from the microarray dataset. Protein and mRNA data were combined in the Microsoft Access query tool. Data were processed by normalizing expression values to reference data and generating ratios of mRNA data from each replicate of the experiment by Partek® Genomics Suite™ software. Ratios were averaged in Excel using the GeoMean (geometric mean) function, and the student’s T-test was performed to obtain *P* values. Hierarchical cluster analysis was performed with Cluster 3.0 software.

### Real-time Quantitative RT-PCR

Total RNA was extracted using Trizol reagent (Gibico BRL, Grand Island, New York, USA) according to the manufacturer’s instructions. Two microgram of RNA per sample was reverse-transcribed into first-strand cDNA by using the A3500 reverse transcription system (Promega, USA) in a standard protocol with random oligo (dT) primers. According to the manufacturer’s instructions, real-time PCR amplifications were performed on the Roche LightCycler system (Roche Diagnostics, Mannheim, Germany) with SYBR Green I dye, which binds preferentially to double-strand DNA and enables real time detection of PCR products. The cDNA was submitted to real-time PCR using the following primer pairs as shown in Table S2 (Supporting Information) (Origene, Rockville, MD). Briefly, a 20 µl reaction mixture containing 2 µl of cDNA, 2 µl of Faststart DNA Master SYBR Green 1 mix (Roche Diagnostics, Mannheim, Germany), 0.5 µl of 10 µmol/L PCR forward primers, 0.5 µl of 10 µmol/L PCR reverse primers, 1 µl of 25 mmol/L MgCl_2_ and 14 µl H_2_O was loaded into glass capillary tubes, and cycling was carried out as follows: 50°C for 2 min and 95°C for 5 min followed by 40 cycles of 95°C for 30 s, 56°C for 30 s and 72°C for 30 s. After each run, the cycle threshold (CT) values were provided by real-time PCR instrumentation by the LightCycler software. A melting curve analysis was performed to determine the specificity of the amplified products. Analysis of relative gene expression was performed using the 2^−ΔΔ*C*T^ method as described [27]. Evaluation of 2^−ΔΔ*C*T^ indicates the fold change in gene expression relative to the internal standard gene *28S* and takes into account the standard deviation. Individual CT values were based on three separate measurements. The specificity of the PCR amplification was directly verified by melt-curve analysis of the final products in the iCycler. To verify the melting curve data, all PCR products were verified by DNA sequencing.

### Western Blot Analysis

Western blot analyses were performed as described previously [28]. The cells were harvested from flasks, washed twice with cold PBS and lysed in a lysis buffer (50 mmol/L Tris, PH7.4, 100 mmol/L NaCl, 1 mmol/L MgCl_2_, 2.5 mmol/L Na_3_VO_4_, 1 mmol/L PMSF, 2.5 mmol/L EDTA, 0.5% Triton X-100, 0.5% NP-40, 5 µg/mL of aprotinin, pepstatin A, and leupeptin) for 60 min on ice, followed by centrifuging at 11,000×g for 15 min at 4°C to remove cell debris. Then, proteins were quantified by the Bradford reagent assay (Bio-Rad). After an addition of 2 × loading buffer, 80 µg of lysate was boiled at 95°C for 5 min and was separated through 10% or 12% SDS-PAGE gels. Proteins were subsequently electrotransferred to Hybond-P PVDF membranes. After blocking with 5% nonfat dry milk in TBS-T containing 0.1% Tween-20 for 2 h at room temperature, the membranes were probed with anti-DNMT3B, anti-CTNNB1, anti-HDAC2, anti-VIM, anti-DNMT3A, anti-NES, anti-HSPA1A, anti-HIST1H1B, anti-H3K9ac3, anti-H3ac, anti-H4ac, anti-H4k12ac or anti-β-ACTIN diluted 1∶1000–1∶2000 overnight at 4°C, followed by incubation in a 1∶2000 dilution of secondary antibodies conjugated to horseradish peroxidase for 1 h at room temperature. Antibodies are summarized in Table S1. Protein bands were detected using the ECL detection system, followed by exposure on Hyperfilm (Amersham Biosciences). All Western immunoblots were performed at least three times. In each experiment, membranes were also probed with anti-β-ACTIN antibody to correct for differences in protein loading. The Image J image analysis system was applied to analyze the strap of Western Blot and to calculate their gray-scale ratio relative to the expression of β-ACTIN.

### Quantification of Gene Copy Number

Genomic DNA was isolated from cells using the Qiagen DNAeasy extraction kit (Qiagen). Genomic DNA (50 ng) was amplified using Roche LightCycler system. Gene copy number was compared by the 2^−ΔΔC(t)^ method, and normalized to the results obtained using the nuclear *β-globin* gene of nuclear as an endogenous reference gene [27], [29]. Individual CT values were based on three separate measurements. All primer sequences used for qPCR are listed (Table S3). Specificity for each primer pair was examined by melting curve functionality and PCR products were verified by DNA sequencing.

### Spontaneous Differentiation of hES Cells

Spontaneously *in vitro* differentiation was conducted through embryoid body (EB) formation. Human ES colonies were mechanically dissociated into small clumps and detached to grow as aggregates in suspension to form embryoid bodies in DFSR medium without bFGF. The medium was changed every 2 days. At day 21, EBs were collected for further analysis.

### Statistical Analysis

Independent sample t- tests between groups were used to evaluate the statistical significance of mean values by using SPSS 18.0 for Windows. Homogeneity of variance was analyzed before the independent sample t-test, in which statistical significance levels of the two variance estimates was *P*>0.1. If the varience was not equal, unequal-variances *t* test was used. Statistical significance levels were *P*<0.05 (denoted as *). All *P* values were two-tailed.

## Results

### Detection and Relative Quantification of Proteins in Abnormal and Normal chHES-3 Cell Lines

Our previous study reported progressive karyotypic changes from simple to complex in *ch*HES-3 cells during a long-term suboptimal culture, which underwent deregulation of self-renewal and dysfunction of related genes and led to malignant transformation [8], [9]. To carry out our quantitative proteomics analyses, *ch*HES-3 cells at different karyotypic stages, were confirmed by karyotype and verified with well-established markers of pluripotency (Figure S1; Supporting Information). The G-banding results showed that the normal *ch*HES-3 cells (passage 30, P30) retained a normal karyotype, SIMP *ch*HES-3 cells (passage 72, P72,) had the karyotype of 46,XX, dup(1)(p32p36) and COMP *ch*HES-3 (passage 182, P182) had a consistent and clonal karyotype of 46, XX, dup(1)(p32p36)t(1;6;4)(q25;q23;p16)ins(4;1)(p16;q21q25),der(2)t(2;7)(q35;qter)t(7;8)(q22;q22),inv(10)(p11q21),der(15)t(4;15)(q21;q26), based on the principle of human cytogenetic nomenclature of ISCN 2013 [30]. Standard G-banding analyses of three successive passages of hESCs showed a consistent and clonal karyotype. The cellular immunohistochemistry data showed that chHES-3 cells were positive for alkaline phosphatase activity, OCT-4, TRA-1-60, TRA-1-81, SSEA-4 and SSEA-3, and were negative for SSEA-1, which confirmed that karyotypically aberrant *ch*HES-3 cells used in this study had basic features consistent with normal *ch*HES-3 cells.

**Figure 1:**
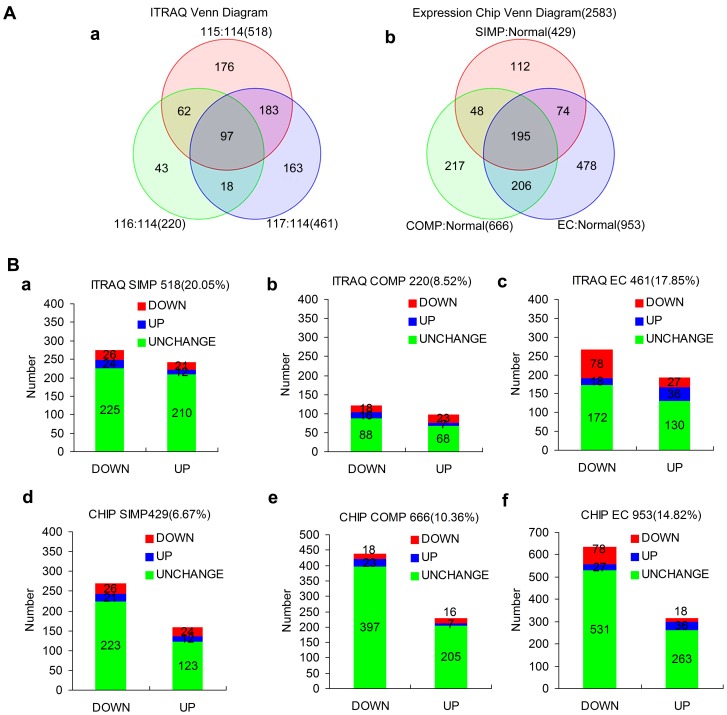
Comparative analysis of transcriptomic and proteomic data in normal and karyotypically aberrant *ch*HES-3 cells. (A) Showing the Venn diagrams of the number of differentially expressed protein (a) and gene probes (b) of 2583 expressed proteins in SIMP (red), COMP (green) *ch*HES-3 cells, and EC cells (blue) as compared with normal *ch*HES-3 cells. (B) Pairwise comparison of the protein (a–c) and mRNA (d–f) ratios between normal *ch*HES-3 cells with karyotypically aberrant *ch*HES-3 cells and EC cells. Microarray and iTRAQ data of 2583 proteins in SIMP as compared normal (a, d); or in COMP as compared normal (b, e) *ch*HES-3 cells; or between EC cells and normal (c, f) *ch*HES-3 cells. Comparative analyses were expressed as being up-regulated or down-regulated or unchanged.

In this study, iTRAQ was used for multiplexed peptide profiling of normal and karyotypically aberrant *ch*HES-3 cells and EC cells labeled with four independent reagents of the same mass that upon fragmentation in MS/MS, gave rise to four unique reporter ions (m/z = 114–117). These were subsequently used to quantify the Normal, SIMP and COMP *ch*HES-3 cells, and EC cells respectively. Examination of the average values and standard deviations of the data from triplicate experiments revealed that the overall variation was less than 30%. LC-MS/MS analysis of 20 SCX fractions from cell lysates generated a total of 190, 569 validated MS/MS spectra. Using a confidence cut-off score, defined as the ProtScore value >1.3 (95% confidence), a total of 2583 proteins were identified from 102, 640 distinct peptides. The iTRAQ ratios, their respective statistical values and % coverage for a total of 2583 proteins detected with 95% confidence are shown in Table S4 (Supporting Information). Nearly, 518 proteins showed more than 1.3 fold changes (ratio <0.77 or >1.3) in expression levels and had *P*<0.05 in SIMP *ch*HES-3 cells as compared to the normal cell line. By contrast, 220 proteins showed more than 1.3 fold changes with *P*<0.05 in COMP *ch*HES-3 cells (Figure 1A-a). Due to space constraints, 316 proteins with more than 1.3 fold changes in SIMP or COMP *ch*HES-3 cells with *P*<0.05 as compared to the normal cell line are shown in Table S5.

**Figure 2:**
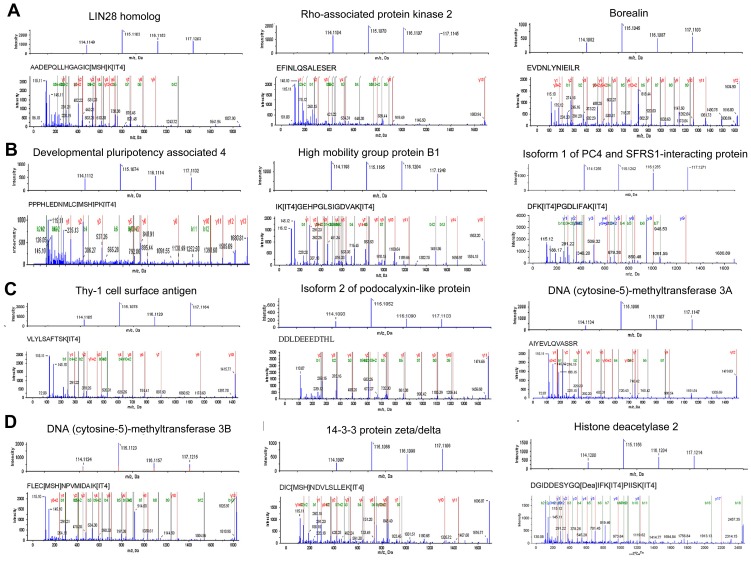
MS/MS spectra of iTRAQ labeled peptides from selected proteins. This shows quantitative data (top panel) and MS/MS spectra (lower panel) of peptides from the LIN28 homolog (LIN28), Rho-associated protein kinase 2 (ROCK2), Borealin (CDCA8), developmental pluripotency associated 4 (DPPA4), high mobility group protein B1 (HMGB1), isoform 1 of PC4 and SFRS1-interacting protein (PSIP1), Thy-1 cell surface antigen (THY1), Podocalyxin-like protein (PODXL), DNA (cytosine-5-)-methyltransferase 3 alpha (DNMT3A), DNA (cytosine-5)- methyl- transferase 3 beta (DNMT3B), 14-3-3 protein zeta/delta(YWHAZ), and histone deacetylase 2 (HDAC2), respectively. The top panel shows the relative peak heights of the iTRAQ labels while the lower panel shows a representative tandem mass spectrum of a peptide in each protein. The reporter ions in the top panels show examples of equal, low and high protein expression in normal and aberrant *ch*HES-3 cells. Normal *ch*HES-3 cells, simple duplication *ch*HES-3 cells, complex karyotypic *ch*HES-3 cells, and EC cells are labeled as 114, 115, 116 and 117 respectively. The x-axis of a mass spectrum represents a relationship between the mass of a given ion and the number of elementary charges that it carries. This is written as the IUPAC standard m/z to denote the quantity formed by dividing the mass of an ion by the unified atomic mass unit and by its charge number (positive absolute value). Data were described in Daltons (Da) as the unit of mass. The y-axis of a mass spectrum represents signal the intensity of the ions.

By contrast, Table 1 provides the partial list of proteins (top 10) identified in this study that were highly expressed or decreased in SIMP or COMP *ch*HES-3 cells, or EC cells when compared with Normal *ch*HES-3 cells. MS/MS and iTRAQ reporter ion spectra of representative peptides from 12 proteins with different expression levels in normal and karyotypically aberrant *ch*HES-3 cells and EC cells were shown in Figure 2. The MS/MS spectra and iTRAQ ratios of peptides from LIN28 homolog (LIN28), Rho-associated protein kinase 2 (ROCK2) and Borealin (CDCA8) showed no significant changes (Figure 2 A). The MS/MS spectra and iTRAQ ratios of peptides from developmental pluripotency associated 4 (DPPA4), high mobility group protein B1 (HMGB1) and isoform 1 of PC4 and SFRS1-interacting protein (PSIP1) were highly expressed in normal *ch*HES-3 cells (Figure 2 B). The MS/MS spectra and iTRAQ ratios of peptides from Thy-1 cell surface antigen (THY1), isoform 2 of podocalyxin-like protein (PODXL) and DNA (cytosine-5)-methyltransferase 3A (DNMT3A) were highly expressed in SIMP *ch*HES-3 cells (Figure 2 C). Furthermore, Panels of Figure 2 D showed MS/MS spectra of peptides from DNA (cytosine-5)-methyltransferase 3B (DNMT3B), 14-3-3 protein zeta/delta (YWHAZ), and histone deacetylase 2 (HDAC2), which were all highly expressed in both SIMP and COMP *ch*HES-3 cells. Quantitative data was supported by *P*-values wherever more than two peptides were used for quantitation, and each was done with biological replicates. The error factor, which was similar to the SD and gave a measure of the certainty of the average ratio, and the number of peptides (>95% confidence) used for quantitation were also included. The ProteinPilot software program calculates the Error factor = 10 ^95%Confidence error^_._

**Table 1:** Partial list of proteins identified to be expressed at different levels between normal and aberrant *ch*HES-3 cells by iTRAQ analysis^a^.

N	Unused	Total	%Cov (95)	Accession	Gene Symbol	Peptides (95%)	115∶114	PVal 115∶114	116∶114	PVal 116∶114	117∶114	PVal 117∶114
2106	2.02	2.02	10.78	IPI:IPI00550020.3	PTMS	2	0.36	1.35E-02	0.63	8.17E-03	0.81	3.46E-01
1307	3.61	3.62	10.94	IPI:IPI00848331.1	RPL31	2	0.42	1.75E-04	0.61	2.81E-03	0.81	3.10E-01
439	11.08	11.1	10.67	IPI:IPI00745872.2	ALB	7	0.43	2.48E-12	0.65	1.34E-10	0.55	1.79E-08
1017	4.73	4.74	13.33	IPI:IPI00014938.3	SARNP	3	0.43	1.52E-03	0.64	2.30E-05	0.58	8.14E-05
167	21.85	21.85	48.57	IPI:IPI00815642.1	TMSB4X	15	0.43	9.33E-29	0.82	1.64E-05	0.74	7.24E-12
2112	2.01	6.46	8.35	IPI:IPI00300763.4	METAP2	3	0.46	6.73E-02	0.57	7.04E-02	0.45	4.98E-02
979	5.01	6.02	21.47	IPI:IPI00024662.1	CBX5	3	0.47	1.70E-02	0.59	5.67E-03	0.55	1.45E-01
149	23.45	23.45	20.35	IPI:IPI00217468.3	HIST1H1B	39	0.48	4.49E-35	0.49	4.49E-29	0.49	3.29E-34
678	7.71	7.71	34.69	IPI:IPI00304409.3	CARHSP1	5	0.49	8.93E-05	0.73	4.28E-03	0.38	1.14E-04
295	14.66	15.07	35.35	IPI:IPI00419258.4	HMGB1	12	0.49	1.67E-21	0.7	4.97E-14	0.45	8.51E-16
356	12.79	12.83	42.19	IPI:IPI00514127.1	HDGF	7	0.5	4.23E-09	0.61	1.07E-06	0.83	5.39E-03
431	11.19	15.26	14.48	IPI:IPI00217466.3	HIST1H1D	12	0.52	1.51E-09	0.55	6.58E-10	0.28	6.11E-12
253	16.45	16.47	29.22	IPI:IPI00219301.7	MARCKS	16	0.52	1.45E-13	0.67	1.44E-09	0.38	4.27E-09
287	15.06	15.07	31.03	IPI:IPI00658097.1	HMGN1	12	0.52	2.00E-09	0.57	3.65E-09	0.72	4.55E-07
146	23.8	23.8	35.68	IPI:IPI00299024.9	BASP1	13	0.53	1.27E-08	0.65	1.18E-06	0.4	8.04E-09
406	11.71	11.72	30.71	IPI:IPI00221222.7	SUB1	6	0.53	5.51E-10	0.61	1.99E-08	0.5	6.97E-08
134	25.68	25.69	30.38	IPI:IPI00028122.1	PSIP1	20	0.56	3.63E-18	0.59	3.57E-17	0.59	4.98E-13
1222	4.01	4.04	14.08	IPI:IPI00783862.2	BLVRB	2	0.58	1.49E-04	0.6	6.77E-04	0.59	5.36E-04
1361	3.4	8.29	40.68	IPI:IPI00030929.4	MYL9	5	0.68	5.26E-03	0.79	8.32E-02	0.27	2.41E-02
329	13.63	13.64	49.26	IPI:IPI00293434.2	SRP14	11	0.69	4.08E-04	0.73	1.51E-03	0.36	9.40E-08
2374	1.57	11.61	40.98	IPI:IPI00793442.1	PHB	9	0.71	7.53E-02	0.61	5.41E-02	2.62	7.80E-02
194	19.53	19.54	41.47	IPI:IPI00022977.1	CKB	19	0.74	2.90E-13	0.85	4.01E-05	0.41	7.84E-21
284	15.13	15.18	43.5	IPI:IPI00604523.1	MYL12A	17	0.75	2.74E-07	1.25	2.35E-04	0.41	1.86E-10
395	12.03	12.04	17.2	IPI:IPI00382990.1	DERP12	6	0.93	2.51E-01	0.9	3.58E-01	4.86	2.81E-04
2067	2.03	2.07	1.52	IPI:IPI00018213.5	LRRC8D	2	1.01	8.78E-01	0.69	7.96E-02	6.58	4.20E-02
1235	4	4.02	21.62	IPI:IPI00792758.1	ARHGDIB	2	1.12	2.40E-01	2.98	8.63E-04	0.39	5.56E-04
1062	4.39	4.4	5.23	IPI:IPI00298971.1	VTN	2	1.12	4.78E-01	1.24	1.65E-01	6.89	9.77E-06
74	35.43	35.45	32.23	IPI:IPI00021405.3	LMNA	28	1.2	2.68E-07	2.06	4.71E-20	1.12	6.26E-02
745	6.8	6.81	20.98	IPI:IPI00025512.2	HSPB1	4	1.2	7.38E-02	1.22	5.56E-02	2.45	7.97E-05
904	5.72	5.72	25.71	IPI:IPI00013895.1	S100A11	8	1.21	4.32E-04	2.15	7.02E-10	1.56	1.42E-08
392	12.06	12.09	60	IPI:IPI00219219.3	LGALS1	9	1.28	6.86E-05	2.33	2.69E-10	0.55	6.63E-05
1243	4	4	39.08	IPI:IPI00436518.1	BRL	3	1.33	5.30E-03	3.28	3.50E-05	0.65	3.33E-02
821	6.17	6.76	13	IPI:IPI00024095.3	ANXA3	3	1.33	1.60E-03	2.3	3.25E-05	0.7	2.24E-04
600	8.58	8.59	17.07	IPI:IPI00101037.3	RCN3	4	1.33	1.86E-02	2.05	8.20E-03	0.87	1.74E-01
1228	4.01	4.02	16.58	IPI:IPI00442073.5	CSRP1	2	1.34	4.90E-03	2.38	1.96E-02	1.26	4.67E-01
169	21.74	21.74	29.04	IPI:IPI00219217.3	LDHB	16	1.38	8.14E-09	0.87	1.16E-06	2.26	4.63E-14
654	8.01	8.02	19.76	IPI:IPI00295741.4	CTSB	7	1.43	2.88E-03	2.71	4.16E-04	0.38	4.67E-03
1795	2.19	2.23	4.95	IPI:IPI00414079.1	ATP6V1H	3	1.47	7.22E-02	1.31	9.95E-02	2.46	1.26E-02
1223	4.01	4.04	16.55	IPI:IPI00555577.1	THY1	2	1.54	2.96E-02	1.12	3.39E-01	2.45	1.00E-02
569	9.03	11.08	27.35	IPI:IPI00018146.1	YWHAQ	6	1.66	3.28E-03	1.22	1.52E-01	3.09	4.96E-05
379	12.23	12.25	19.56	IPI:IPI00020632.4	ASS1	6	1.73	6.24E-03	2.28	8.09E-03	1.11	7.04E-01
2179	2	2.01	8.78	IPI:IPI00397358.4	RPS27	2	1.83	3.45E-03	2.09	1.11E-05	1.62	8.10E-05
301	14.56	15.77	32.76	IPI:IPI00455531.2	VDAC2	10	2.19	3.76E-07	1.4	6.93E-05	1.72	1.18E-05
1041	4.53	4.54	7.67	IPI:IPI00020944.1	FDFT1	2	2.2	1.04E-02	1.3	1.43E-02	2.5	1.94E-03
668	7.84	7.84	18.05	IPI:IPI00005719.1	RAB1A	3	2.21	1.07E-02	1.55	4.05E-04	1.48	1.73E-01
818	6.19	6.2	10.62	IPI:IPI00029046.1	MLEC	5	2.26	7.97E-06	1.32	4.50E-02	2.36	4.23E-03
1205	4.03	4.07	15.97	IPI:IPI00018871.2	ARL8B	2	2.39	4.08E-02	1.26	1.16E-01	1.29	5.42E-01
1362	3.4	4.01	12.02	IPI:IPI00018364.2	RAP2B	2	2.48	2.09E-02	1.47	2.77E-02	2.17	3.62E-03
1148	4.13	4.16	12.68	IPI:IPI00873632.1	RAB2A	2	2.66	2.53E-02	1.46	1.27E-01	1.93	2.56E-02
1204	4.03	4.07	5.5	IPI:IPI00005745.1	SPTLC1	2	2.67	1.39E-01	1.75	1.25E-02	2.79	1.99E-01
1122	4.2	4.2	15.17	IPI:IPI00871870.1	ARPC3	2	2.72	1.12E-03	1.37	4.08E-02	1.15	3.55E-01
894	5.79	5.8	20.35	IPI:IPI00909530.1	H3F3B	5	3.72	5.27E-03	1.06	5.47E-01	1.07	1.78E-01

*a* Only the top 10 up- and down-regulated proteins found to be highly expressed or decreased in SIMP and COMP *ch*HES-3 cells, and EC cells as compared with normal *ch*HES-3 are shown.

Note that 115∶114, 116∶114, and 117∶114 refer to relative levels of protein expression in SIMP *ch*HES-3, COMP *ch*HES-3 and hECCs cells with respect to normal *ch*HES-3 cells. Statistical calculations for iTRAQ-based detection and relative quantification were calculated using the Paragon Algorithm using Protein-Pilot software.

### Comparison between the Proteome and Transcriptome of Normal and Karyotypically Aberrant chHES-3 Cells

Further analysis of these data was performed by comparing relative quantification of proteins with the microarray data. We correlated 2583 proteins with probe sets from the microarray analysis. The reason a complete comparison was not made was because of an inability of the Partek® Genomics Suite™ software (Gene Company Limited) to match certain protein accession numbers to their associated microarray probe sets with a sufficiently high level of certainty. We thus concentrated data analysis where co-identity of protein and probe set data was assured (Table S6). Based on an analysis of the matched datasets we found the vast majority of identified proteins and mRNA were unaltered, and a low correlation between the protein and microarray changes seen for each gene product (Figure 1). This was in agreement with several other proteomic analyses in multiple systems, which have concluded that the proteome and transcriptome correlate only weakly, if at all [31]–[36].

Venn diagram illustrations of the data showed protein expression level changes by ITRAQ analysis (a) and mRNA level changes by expression chip analysis (b) for both SIMP and COMP *ch*HES-3 cells, and for EC cells as compared with normal *ch*HES-3 cells (Figure 1A). The SIMP (iTRAQ 115) *ch*HES-3 cells, COMP *ch*HES-3 cells (iTRAQ 116), and EC cells (iTRAQ 117) as compared with normal (iTRAQ 114) *ch*HES-3 cells, showed 518(20.05%), 220(8.52%) or 461(17.85%) differentially expressed proteins respectively, with common proteins in 97 cases (Figure 1A-a, Figure 1B). With regard the changes in mRNA levels of 2583 identified proteins by ITRAQ analysis, the results obtained from the changes in expression as shown by chip probe analysis, showed changes in 429, 666 or 953 probe values from SIMP *ch*HES-3 cells, COMP *ch*HES-3 cells, and EC cells respectively, with common probes in 195 cases (Figure 1A-b).

However, many changes in protein levels could not be ascribed to altered mRNA expression based on the lack of correlation spanning each pair-wise comparison of the protein and mRNA ratios between the normal and aberrant karyotypic hESCs (Figure 1B). The SIMP (iTRAQ 115) *ch*HES-3 cells (Figure 1B-a) showed 210 cases (of a total of 518 protein changes) as compared with normal *ch*HES-3 cells (iTRAQ 114) where the proteins were significantly augmented, yet mRNA levels were not significantly changed. In addition, in 225 cases where the proteins were significantly down-regulated, we did not shown any significantly changed mRNA expression levels as compared to normal *ch*HES-3 cells (iTRAQ 114). Only 38 (12 upregulated and 26 down-regulated) of the 518 protein changes correlated with similar changes seen in mRNA levels. This trend continued when the COMP (iTRAQ 116) *ch*HES-3 cells (Figure 1B-b) were compared with normal *ch*HES-3 cells (iTRAQ 114), which showed 68 cases (of a total of 220 protein changes) where the proteins were significantly enhanced, yet the mRNA levels were not significantly changed. In addition, there were 88 cases where the proteins were significantly down-regulated yet the mRNA levels were not significantly changed. Only 25 (7 upregulated and 18 down-regulated) of the 220 protein changes were seen at the mRNA level as well. As for the EC (iTRAQ 117) cells (Figure 1B-c) as compared with normal *ch*HES-3 cells (iTRAQ 114), this comparison showed 130 cases (of a total of 461 protein changes) where the proteins were significantly upregulated yet the mRNA levels were not significantly changed. Moreover, 172 cases were identified where the proteins were significantly down-regulated yet the mRNA levels were not significantly changed. Finally, 114 (36 upregulated and 78 down-regulated) of the 518 protein changes were correlated to the same changes seen in mRNA.

Furthermore, when changes in mRNA expression levels of 2583 proteins were considered in the SIMP *ch*HES-3 cells as compared with normal *ch*HES-3 cells, 346 genes (123 upregulated and 223 down-regulated) of a total of 429 with changes in mRNA expression were identified; in these cases, levels of protein expression were found to be unaltered (Figure 1B-d). As for COMP *ch*HES-3 cells, 602 (205 upregulated and 397 down-regulated) of a total of 666 genes with mRNA expression changes were identified; in these cases, protein expression levels were unaltered as compared with normal *ch*HES-3 cells (Figure 1B-e). Finally, there were 794 genes (263 upregulated and 531 down-regulated) of a total of 953 in EC cells showing changes in mRNA expression levels; in this analysis, the protein expression levels were unaltered as compared with normal *ch*HES-3 cells.

**Figure 3:**
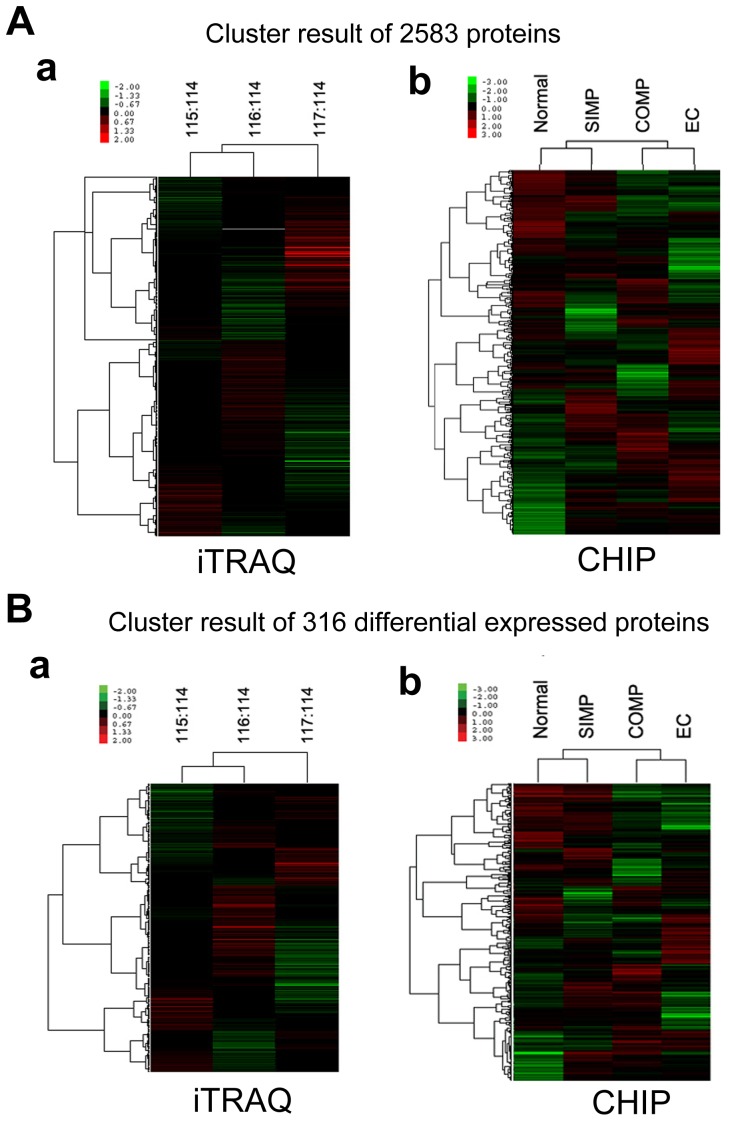
Cluster analyses of transcriptomic and proteomic data in normal and karyotypically aberrant *ch*HES-3 cells. (A) Cluster analyses of 2583 gene product ratios measured at the protein level by iTRAQ analysis (a), and mRNA levels by expression of chip data (b) in normal (iTRAQ 114), SIMP (iTRAQ 115), COMP *ch*HES-3 cells (iTRAQ 116) and NTERA-2 (ITRAQ 117). Only the gene products that were observed at the protein and mRNA levels were included in this figure. (B) Cluster analyses of iTRAQ (a) and expression chip data (b), of 316 differentially expressed proteins among Normal (iTRAQ114), SIMP (iTRAQ115) and COMP *ch*HES-3 cells (iTRAQ116).

However, hierarchical cluster analysis of 2583 proteins datum (Figure 3A) in ITRAQ analysis (a), and in associated microarray probe sets (b), showed that Normal *ch*HES-3 cells and SIMP *ch*HES-3 cells were more closely related to each other than to COMP *ch*HES-3 cells, which were more closely related to EC cells. Coincident with the cluster results of a total number of 2583 proteins, hierarchical cluster analysis of 316 differential expressed proteins (Figure 3B) in ITRAQ analysis (a) and in associated microarray probe sets (b), showed similar observations. But, studies of protein levels in these cells were also required since the levels of protein expression do not always directly correlate with the transcriptomic changes (Figure 1A–B).

### Categorization and Functional Annotation Analysis of Proteins Quantitated in Normal and Aberrant chHES-3 Cells

**Figure 4:**
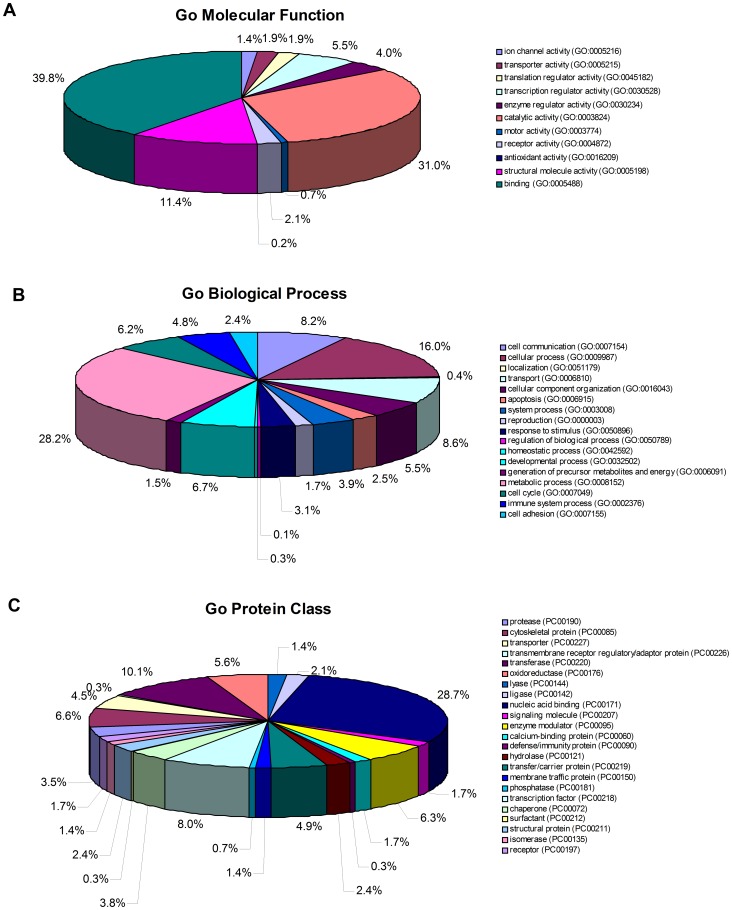
Functional annotation of differential proteins identified from normal *ch*HES-3 cells and karyotypically aberrant *ch*HES-3 cells. The pie chart shows the various statistically significant (at *P*<0.05) functional categories as a percentage of the 316 differentially expressed proteins based on the PANTHER classification system, and proteins justifying specific biological functions.

Functional annotations of the combined lists of proteins from all three experiments are shown in Figure 4. These 316 proteins, which were differentially expressed between normal and aberrant *ch*HES-3 cells, could be classified into 11 functional categories using the PANTHER classification system (http://www.pantherdb.org). The top three molecular function categories were binding (GO:0005488)(39.8%), catalytic activity (GO:0003824) (31.0%), and structural molecule activity (GO:0005198) (11.4%). Futhermore, significantly over- and under-represented GO biological process and protein class terms for the set of differentially expressed proteins were represented as follows: the top three biological process categories were metabolic process (29.0%), cellular process (16.5%) and cell communication (8.0%). Examples of some early developmental markers high in normal karyotypic hESCs included NES (Nestin), GRB2 (Isoform 1 of Growth factor receptor-bound protein 2), RBM14 (Isoform 1 of RNA-binding protein 14) and HNRNPAB (Heterogeneous nuclear ribonucleoprotein A/B). Several proteins related to hyper-proliferation and suppression of apoptosis such as HDAC2 and API5 (Apoptosis inhibitor 5) were highly expressed in abnormal hESCs. However, proteins that were associated with apoptosis such as HNRNPK (Heterogeneous nuclear ribonucleoprotein K), DFFA (DNA fragmentation factor subunit alpha) and PRKDC (DNA-dependent protein kinase catalytic subunit) were down-regulated in the karyotypically abnormal hESCs.

In addition, several proteins that were highly expressed in abnormal hESCs such as DNMT3B, DNMT3A, KPNB1 (Importin subunit beta-1), RAP2B (Ras-related protein Rap-2b), CSNK2A1 (Casein kinase II subunit alpha), PRDX1 (Peroxiredoxin-1), VDAC2 (voltage-dependent anion channel 2), and YWHAZ have also been previously attributed to malignancy in other systems [18], [37]–[41]. By contrast, the expression levels of 316 proteins representing at least 23 protein class categories including chaperone, kinases, proteases, ligase, calcium binding, nucleic acid binding and cytoskeletal proteins were found to be significantly different when comparing normal and abnormal hESCs. The top three protein class categories were nucleic acid binding proteins (28.7%), transferases (10.1%) and transcription factors (8.0%).

### Real-time Quantitative RT-PCR and Western Blot Analysis Validation of Differentially Expressed Proteins

**Figure 5:**
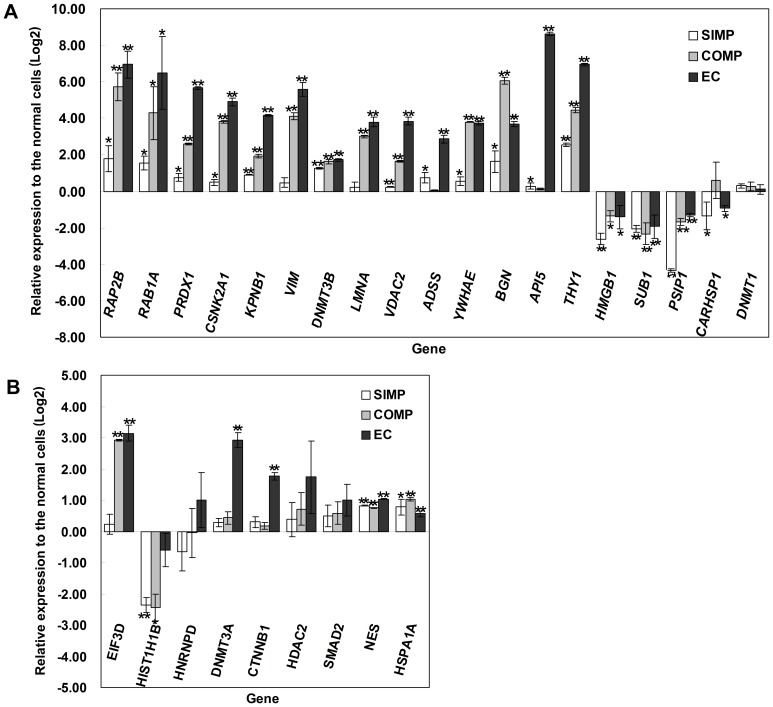
Real-time quantitative RT-PCR analysis to validate differentially expressed proteins. (A) Real-time quantitative RT-PCR validation of mRNA expression levels of 19 differentially expressed proteins consistent with both transcriptomic and proteomic analysis in SIMP and COMP *ch*HES-3 cells when compared with the Normal *ch*HES-3 cells. (B) Real-time quantitative RT-PCR validation of mRNA expression levels of 9 differentially expressed proteins, which were not consistent with proteomic analysis. Data are represented as mean ± S.D. (n = 3). *P<0.05 with respect to the genes expression in Normal *ch*HES-3 cells by t-test.

**Figure 6:**
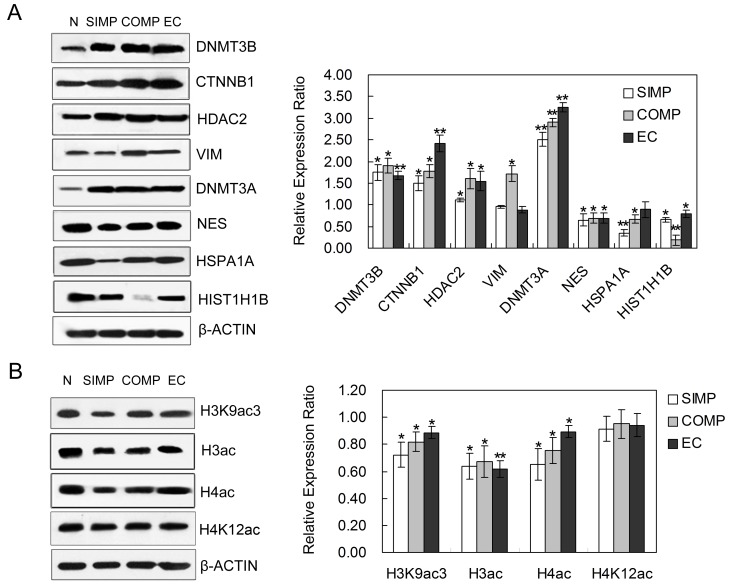
Western blot analysis to validate differentially expressed proteins. (A) Western blot analysis to validate differentially expressed proteins in Normal (N), SIMP and COMP *ch*HES-3 cells. Gray-scale ratios relative to the expression of β-ACTIN in SIMP and COMP *ch*HES-3 cells were compared with the ratio of Normal *ch*HES-3 cells. (B) Western blot analysis showing decreased acetylation levels of histones including H3, H4 and H3K9 in karyotypically aberrant *ch*HES-3 and EC cells, with no change of H4K12. Data are represented as mean ± S.D. (n = 3). *P<0.05, **P<0.01 with respect to the protein expression in Normal *ch*HES-3 cells by independent sample t tests.

To corroborate the proteomic analyses, the relative expression levels of selected proteins that were differentially expressed, were also compared by real-time quantitative RT-PCR (Figure 5) and Western blot analysis (Figure 6). Real-time quantitative RT-PCR showed that the relative expression levels were consistent with both transcriptomic and proteomic analysis for the following 18 proteins: RAP2B, RAB1A (Isoform 1 of Ras-related protein Rab-1A), PRDX1, CSNK2A1, KPNB1, VIM (Vimentin), DNMT3B, LMNA (Isoform A of Lamin-A/C), VDAC2, ADSS (Adenylosuccinate synthetase isozyme 2), YWHAE (14-3-3 protein epsilon), BGN (Biglycan), API5, THY1, HMGB1, SUB1 (Activated RNA polymerase II transcriptional coactivator p15), PSIP1 (Isoform 1 of PC4 and SFRS1-interacting protein), CARHSP1 (Calcium-regulated heat stable protein 1) and DNMT1 (DNA (cytosine-5)-methyltransferase 1) (see Figure 5A).

We also investigated relative mRNA expression levels of EIF3D (eukaryotic translation initiation factor 3, subunit D), HIST1H1B (Histone H1.5), HNRNPD (AU-rich element RNA binding protein 1, P37 kDa) were consistent with proteomic analysis, while they showed no effective signaling in the analysis of the expression chip datum (Figure 5B). Furthermore, some genes such as DNMT3A, CTNNB1 (β-catenin), HDAC2, SMAD2,NES, and HSPA1A (heat shock 70 kDa protein 1A) showed no significant changes in mRNA expression levels, whereas the proteomic analysis showed that these proteins were differentially expressed in karyotypically aberrant *ch*HES-3 cells as compared with normal *ch*HES-3 cells (Figure 5B).

To further verify the proteomic data, we studied expression levels of 8 proteins by Western blot analysis, among which DNMT3B, CTNNB1, HDAC2, VIM, and DNMT3A were upregulated in karyotypically aberrant *ch*HES-3 cells or EC cells, while by contrast NES, HSPA1A and HIST1H1B were downregulated. As a loading control, β-ACTIN showed no obvious changes (Figure 6A). Among these, enhanced expression of HDAC2 protein was observed in karyotypically aberrant *ch*HES-3 and EC cells. HDAC2 is a member of histone deacetylases and plays an important role as a transcriptional repressor [42], [43] by interacting with sumoylated substrates, and thus could be associated with the decrease of acetylation levels of histones. So we also investigated the decreased acetylation levels of histones including H3, H4 and H3K9, which was detectable with antibodies recognizing acetylated histone H3 (H3ac), acetylated histone H4 protein (H4ac) and acetylated histone H3K9 (H3K9ac3), with no obvious changes in acetylated histone H4K12 (H4K12ac)(Figure 6B). The balance of histone acetylation is important for proper cellular function, and such changes might malignant formation of karyotypically aberrant *ch*HES-3 and EC cells.

### Copy Number Variations and Functional Analysis of Differentially Expressed Proteins Including DNMT3B, VIM, CTNNB1 and HDAC2

**Figure 7:**
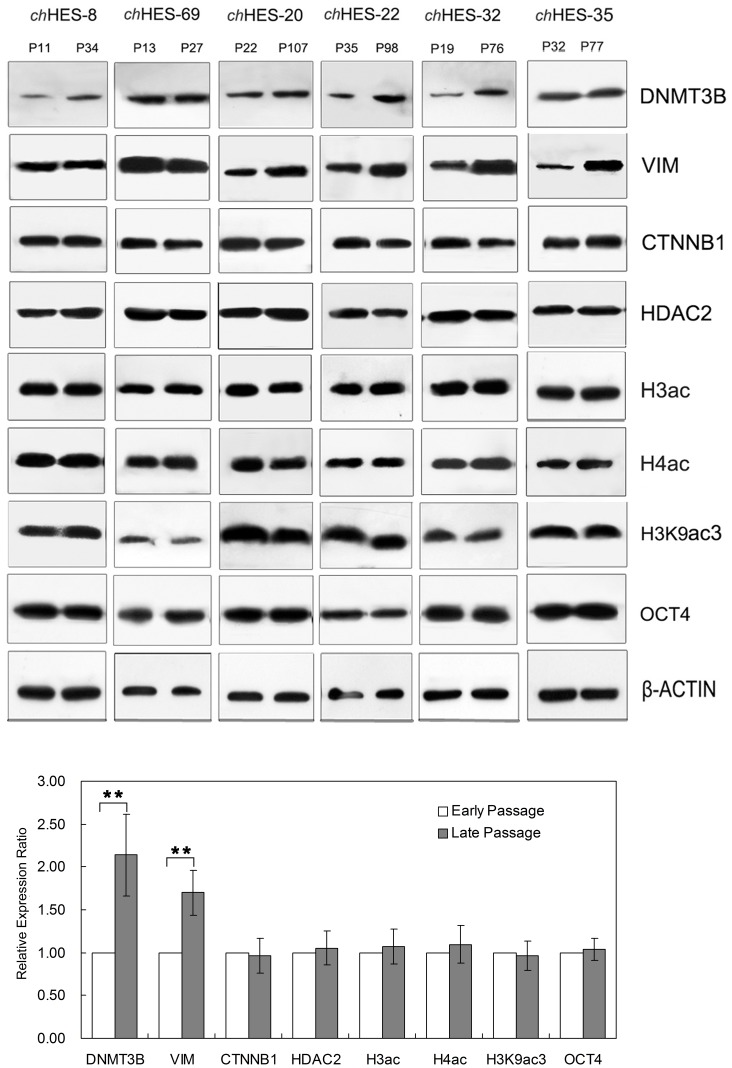
Western blot analysis to validate differentially expressed proteins in early and late passage hESCs. Differentially-expressed proteins and proteomic analysis in the same cell line at early and late passages (*ch*HES-8 P11/P34, *ch*HES-69 P13/P27, *ch*HES-20 P22/P107, *ch*HES-22 P35/P98, *ch*HES-32 P19/P27, *ch*HES-35 P32/P77) were compared. *P* represents for the hESCs culture passage *in vitro*. The late-passage hESCs are over 15∼20 passages prolonged culture in optimal culture conditions after the early–passage hESCs. Data are represented as mean ± S.D. (n = 6). *P<0.05, ** *P*<0.01 with respect to the protein expression in six early passages of hESC lines by independent sample t tests.

This large-scale quantitative proteomic analysis of normal and aberrant karyotypic hESCs should serve as a useful reference set in understanding the malignant transformation process of aberrant karyotypic hESCs which is closely related to changes in DNA methylation, histone acetylation, cell cycle and apoptosis, WNT pathways, and other systems. To verify the iTRAQ data, and to study whether the differentially expressed proteins in the malignant transformation process were vulnerable during prolonged culture under optimal culture conditions [44], Western blot analyses were performed on selected candidates of interest, including DNMT3B, VIM, CTNNB1 and HDAC2. We compared samples of the same line at different passage (*ch*HES-8 P11/P34, *ch*HES-69 P13/P27, *ch*HES-20 P22/P107, *ch*HES-22 P35/P98, *ch*HES-32 P19/P76, *ch*HES-35 P32/P77) (Figure 7), all of which showed a normal karyotype (data not shown). Western blot analyses showed that both DNMT3B and VIM exhibited relatively significant increases in late passages, which showed 2.1 and 1.7 fold changes as compared with early passage of hESCs (*P*<0.01). By contrast, the protein expression of both CTNNB1 and HDAC2 displayed no obvious changes, which was consistent with no obvious change seen in the levels of histone acetylation including H3, H4 and H3K9. Furthermore, OCT4 and β-ACTIN were detected as a loading control, and showed no obvious change in expression (Figure 7, Figure S2).

**Figure 8:**
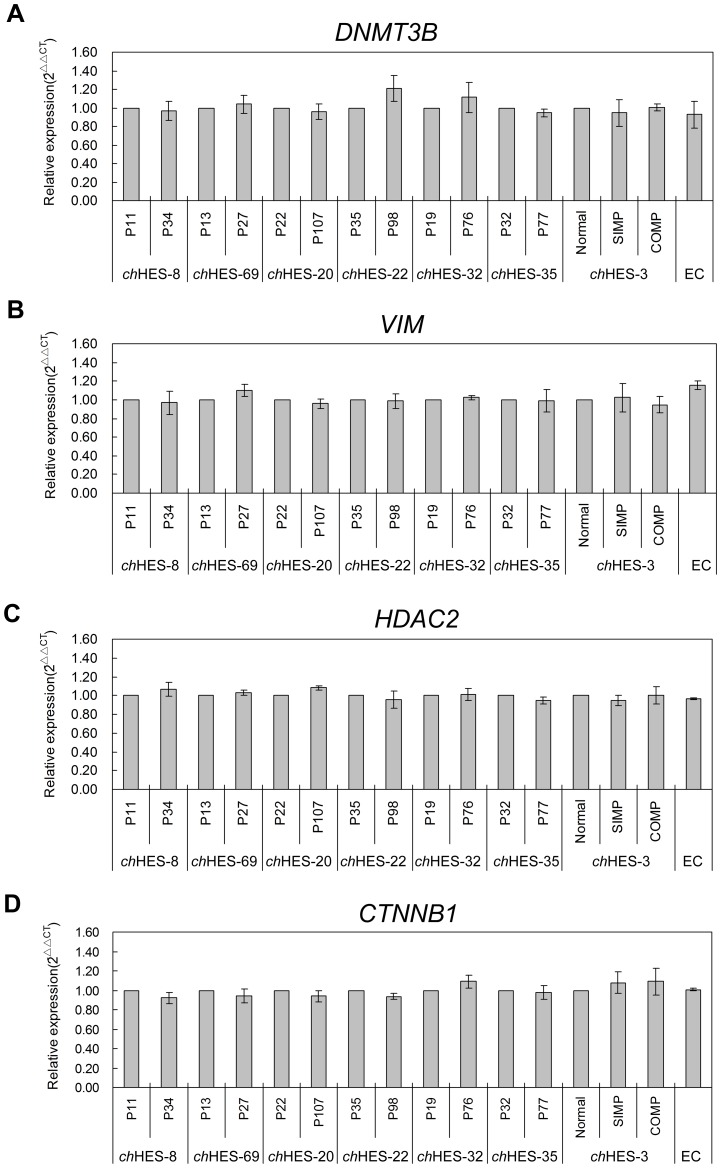
Copy number variation comparison of four differentially expressed genes. Quantification of *DNMT3B*, *VIM*, *CTNNB1* and *HDAC2* in abnormal and normal *ch*HES-3 cell lines, EC cells and six pairs of hESC lines at early and late passage (*ch*HES-8 P11/P34, *ch*HES-69 P13/P27, *ch*HES-20 P22/P107, *ch*HES-22 P35/P98, *ch*HES-32 P19/P27, *ch*HES-35 P32/P77). Gene copy number was compared with β-globin gene of nuclear as an endogenous reference gene. P represents for the hESCs culture passage in vitro. Data are represented as mean ± S.D. (n = 3). Copy number in the genes area of *DNMT3B*, *VIM*, *CTNNB1* and *HDAC2* were showed no significant difference in the same cell line at early and late passages or in abnormal and normal *ch*HES-3 cell lines by independent t-test.

**Figure 9:**
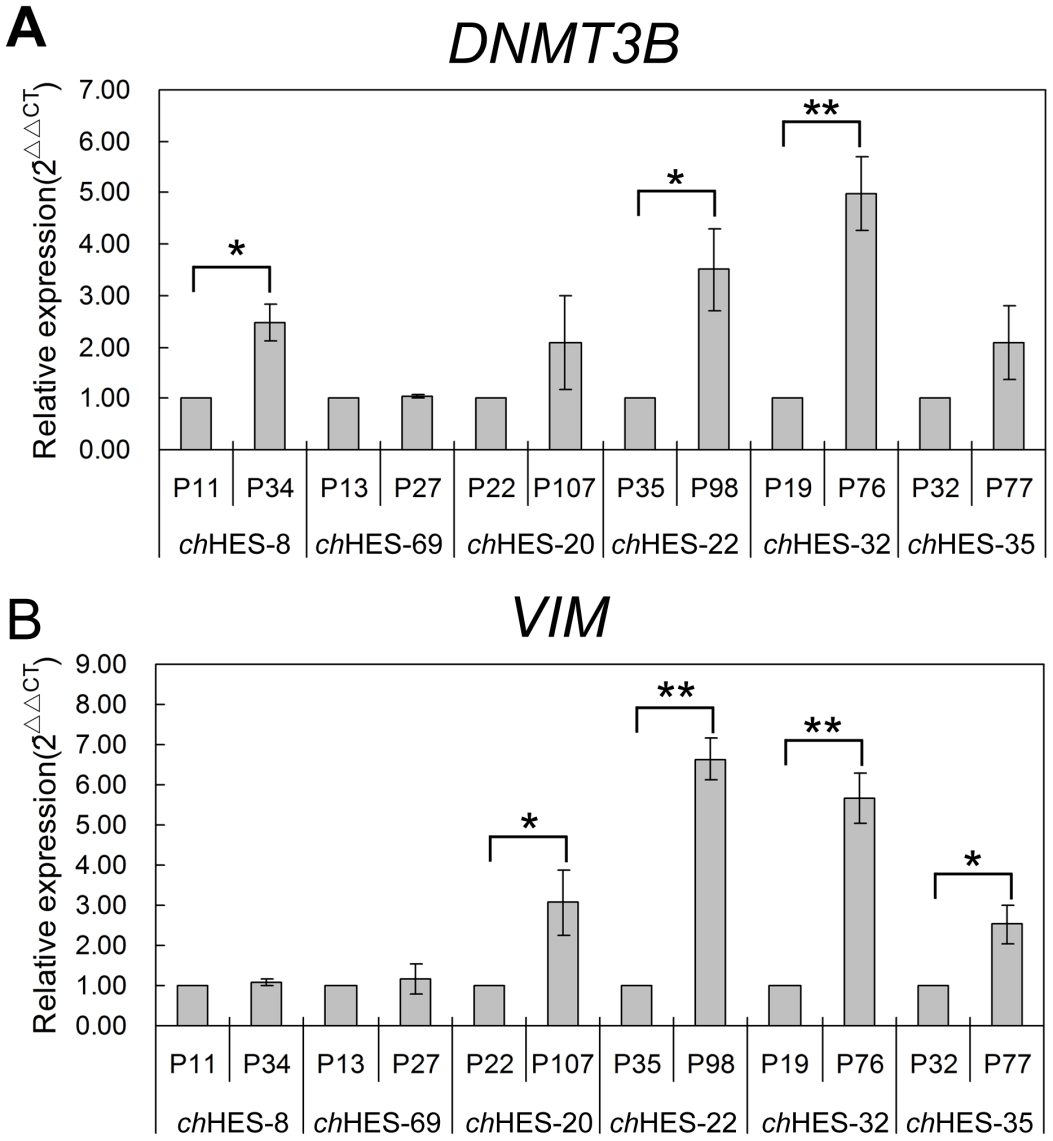
Real-time quantitative RT-PCR analysis of *DNMT3B* and *VIM* expression levels in six pairs of hESCs. Real-time quantitative RT-PCR analysis of *DNMT3B* (A) and *VIM* (B) expression levels in six pairs of hESC lines at early and late passage (*ch*HES-8 P11/P34, *ch*HES-69 P13/P27, *ch*HES-20 P22/P107, *ch*HES-22 P35/P98, *ch*HES-32 P19/P27, *ch*HES-35 P32/P77). P represents for the hESCs culture passage in vitro. Data are represented as mean ± S.D. (n = 3). *P<0.05, **P<0.01 with respect to the mRNA expression in six early passages of hESC lines by independent sample t tests.

Gene amplification is a common mechanism for over-expression of oncogenes in cancers. We thus detected the copy number variation of these genes with up-regulated protein expression, including *DNMT3B*, *VIM*, *CTNNB1* and *HDAC2*, in *ch*HES-3 and six pairs of hESCs samples at different passages, as mentioned above. We did not detect the copy number gain or loss in the amplified gene regions of *DNMT3B*, *VIM*, *CTNNB1* and *HDAC2* by quantitative PCR in late passages of all six hESCs samples and the normal and aberrant karyotypic *ch*HES-3 cells (Figure 8). However, we detected increased mRNA expression of *DNMT3B* and *VIM* in those hESC lines (Figure 9) that demonstrated up- regulation of corresponding protein in the late passages (Figure 7 and Figure S2). Those indicated that increased protein expression of DNMT3B and VIM in late-passage cells might result from the transcriptional regulation, and epigenetic mechanisms, but not copy number amplification.

By contrast, several studies have suggested that the maintenance of mutipotential differentiation ability in embryonic stem cells is controlled by epigenetic mechanisms such as DNA methylation and histone modifications [45]. To determine whether increased expression of genes associated with epigenetic modification such as DNMT3B in the late passages of hESCs affects the pluripotency of hESCs, we compared the differentiation capability of early and late passages hESCs from three cell lines including *ch*HES-8, *ch*HES-69 and *ch*HES-22. All three cell lines were able to differentiate into the three germ layers in vitro. This is consistent with most published studies, which reported that late-culture hESCs still maintained their multi-lineage differentiation ability [46]. However, the expression levels of 8 marker genes representing different germ layers, showed obvious differences among different cell lines rather than the difference between early and late passage of the same cell line. *ch*HES-8 and *ch*HES-69 cells showed decreased expression of most of the marker genes in late passage cells. By contrast, *ch*HES-22 cells showed increased expression levels of most of marker genes in the late passage. But there was no correlation of differences in this differentiation pattern with differentially increased expression levels of *DNMT3B* and *VIM* in the late passage of *ch*HES-8 and *ch*HES-22 cells, while *ch*HES-69 cells showed no clear difference of *DNMT3B* and *VIM* (see Figure S3; Supporting Information). Based on the current experiments, we did not find any differentiation defects or bias for the late-passage hESCs with increased expression of the biomarker genes.

**Figure 10:**
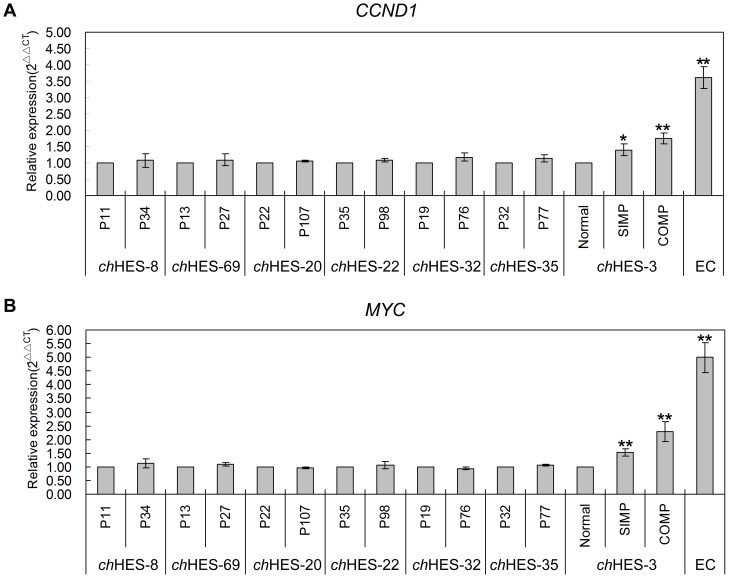
Real-time quantitative RT-PCR analysis of the downstream genes of CTNNB1. Real-time quantitative RT-PCR analysis of *CNND1*(A) and *MYC* (B) expression levels in normal and abnormal *ch*HES-3 cell lines, EC cells and six pairs of hESC lines at early and late passage (*ch*HES-8 P11/P34, *ch*HES-69 P13/P27, *ch*HES-20 P22/P107, *ch*HES-22 P35/P98, *ch*HES-32 P19/P27, *ch*HES-35 P32/P77). P represents for the hESCs culture passage in vitro. Data are represented as mean ± S.D. (n = 3). *P<0.05, **P<0.01 with respect to the expression in normal *ch*HES-3 cell or six early passages of hESC lines by independent sample t tests.

As for CTNNB1 (β-Catenin), it is a major transcriptional modulator of the WNT signal transduction pathway, and showed increased expression in karyotypically abnormal hESCs relative to normal cells. To determine whether elevated CTNNB1 leads to more activated WNT pathway, we analyzed the expression of *MYC* and *CCND1* (*Cyclin D1*), as the downstream genes of CTNNB1, in *ch*HESC-3 and six hESC cell lines at early and late passages. We detected the increased expression of these genes in aberrant *ch*HES-3 but not in six pairs of hESC cells, which are correlated to the expression variation of CTNNB1 protein in these samples (Figure 10). This indicated that the function of CTNNB1 was to modulate the activity of the WNT signaling pathway, and implicated its role in stem cell-derived tumor initiation and progression.

## Discussion

Quantitative proteomics have been shown to be a useful technique for studying the molecular mechanisms in different stages of disease. Relative and absolute quantification (iTRAQ) analysis is currently one of the most widely used approaches for high throughput protein quantitation, and enables simultaneous quantitation of up to 8 different biological samples [47], [48]. The aim of this iTRAQ proteomic study was to gain insight into the molecular mechanisms of the transition of normal embryonic stem cells and provide a screening tool to detect abnormal cells, which would be essential for developing clinical therapies.

Here, we have identified and relatively quantified 2583 proteins with 95% confidence (Table S4) in *ch*HES-3 cell lines of progressive karyotypic change. Hierarchical cluster analysis based on quantified proteins and associated microarray probes demonstrated that *ch*HES-3 cells showed a tendency to malignant transformation from normal *ch*HES-3 cells to COMP *ch*HES-3 cells, which were closely related to EC cells (Figure 3). This further supported that our model successfully simulated the tumorigenic process.

Many factors expressed in early development and those associated with pluripotency were identified, which provides a model system to distinguish pluripotency and oncogenesis. *ch*HES-3 cells at different karyotypic stages were verified with well-established markers of pluripotency including OCT-4, TRA-1-60, TRA-1-81, SSEA-4 and SSEA-3 (Figure S1), which were expressed at such low abundance as to be still detected. However, other more abundant members were expressed in all of the hESCs and hECCs cells. Several of these are known markers of undifferentiated hESC which had been previously reported [18]. Comparing hESCs and hECCs using proteomic analysis, such as analysis of the expression of LIN28, THY1, DPPA4, alkaline phosphatase (ALPL), DNMT3A and DNMT3B, showed 0.99-, 1.54-, 0.88-, 1.28-, 1.51- and 2.18-fold changes in SIMP cells, 0.97-, 1.12-, 0.76-, 0.97-, 1.09- and 1.39-fold changes in COMP cells, and 0.90-, 2.45-, 0.81-, 0.68-, 1.33- and 1.15-fold changes in EC cells, when compared with normal hESCs respectively. However, some relative protein expression levels in different stage of karyotypically abnormal hESCs have not been reported until now. These included SOX2, ROCK2, CDCA8 and GDF7, which showed 0.58-, 1.04-, 0.99- and 0.92-fold changes in SIMP cells, 0.68-, 0.99-, 1.00- and 0.81-fold changes in COMP cells, and 0.52-, 1.15-, 1.04- and 1.09-fold changes in hECCs, as compared with normal cells respectively. Our report demonstrates that these regulators of pluripotency could be associated with early development, and have implications in regulating stem cell destiny.

Microarray analysis is wildly used in the detection of gene expression variations in pre-neoplastic stages to assist in the identification of reliable markers of tumorigenesis at the mRNA level. However, only a few comparisons have been performed on the transcriptome and their associated levels of protein expression [8], [17], [18]. The biological and analytical impact of this comparative analysis demonstrates that changes at the mRNA level cannot be used to assume concordant protein expression levels. Further, it indicates the importance of investigating directly the differences in protein expression. Here, hESCs at a specific stage of transformation were collected to undertake both mRNA and proteomic analyses. There was evidence of a poor statistical agreement between mRNA and protein expression changes for a variety of gene products in karyotypically abnormal hESCs (Figure 1). Modulation of transcription does not directly govern the levels of many proteins, which was also confirmed by the inconsistent results of real-time quantitative RT-PCR validation of differentially expressed proteins in this study (Figure 5B).

We developed an interaction network of proteins identified as changing in expression during transformation of hESCs. DNA methylation and histone deacetylation are key factors in the regulation of transcription. Deregulation of epigenetic information with pluripotent potential may also alter the defining properties of stem cells, their self-renewal and their differentiation potential, leading to the initiation of cancer. An interesting point was found with respect to HDAC2, which belongs to the histone deacetylase family and showed increased expression in karyotypically abnormal hESCs and in EC cells, which was accompanied by decreased acetylation of histones H3, H4 and H3K9 (Figure 6). It is well established that modifications of histones regulate the architecture of chromatin, which is an important factor in regulating gene expression. HDAC2 can interact with HDAC1, and together form the catalytic core of a number of complexes, which target chromatin by sequence-specific transcription factors to repress transcription and in cooperation with other chromatin modifiers. HDAC1/2 could directly mediate repressive functions of a number of well-characterized cellular oncogenes and tumor-suppressor genes, leading to aberrant gene expression [42], [49]. Aberrant regulation of HDAC2 was reported to play a pivotal role in the generation and development of many type of cancers including hepatocellular carcinoma [50], ovarian cancer [37], gastric cancer [51] and others. Giudice [52] reported that chemical inhibition of HDACs reduces the number of cancer stem cells (CSC) and inhibits clonogenic sphere formation. That ablation of HDAC family in many tumor cell lines led to severe proliferation defects or enhanced apoptosis, also suggests that it might be a target for cancer therapy.

We further compared the significance of differentially expressed proteins in our early and later passage hESCs in an optimized culture condition [44] since we wished to exclude the influence of other changes associated with the in vitro culture adaptation. Our data showed relatively stable expression of HDAC2 in long-term in vitro cultures of normal hESCs (Figure 7), but displayed increasing levels during tumorigenesis followed with increased histone deacetylation (Figure 6). These observations supported the idea that enhanced HDAC2 expression may be associated with malignant transformation by regulating the architecture of chromatin. Thus HDAC2 could serve as a potential marker for abnormal hESCs with a tendency of initiating progression to the malignant state.

Also, our data showed increased levels of proteins associated with DNA methylation, including DNMT3A, DNMT3B, DNMT1 and KPNB1. These proteins might contribute to the increased methylation of CpG islands and silencing of affected target genes, which are frequently found in human cancer [53]. Though DNMT3B increases in karyotypically abnormal hESCs, in much the way as HDAC2, expression of DNMT3B was also enhanced in long-term in vitro and optimal culture condition (Figure 7), which was consistent with the gene mRNA expression levels (Figure 9). Recent studies have also shown that DNA methyltransferases, and DNMT3B, were correlated with HDAC1 and HDAC2 and were involved in the epigenetic regulation by silencing transcription [54] and promoting cell proliferation and tumorigenesis [29]. DNMT3B can also interact with HDACs 1/2 and other components of the epigenetic machinery to establish the chromatin environment [55]. Peter Andrews reported that hESCs might undergo culture adaptation in long-term in vitro culture and that variations in gene expression might reflect the aberrant karyotype of the cells or might result from karyotypically silent epigenetic changes, implying that adaptation reflects an alteration in the balance between self-renewal and differentiation [56]. Long-term in vitro cultured hESCs show a high risk of genomic instability due to culture conditions. Thus, we optimized our culture system including the preparation of feeder cells by irradiation, control of the density of feeder cells and passage by manual cutting [44]. In optimized conditions, hESCs show a stable normal karyotype, even after more than two years of in vitro culture. The increased expression of DNMT3B in culture suggested that it might be relevant to culture adaptation and reflect the progressive adaptation of self-renewing cells to their culture conditions. However, the possibility of tumorigenesis can not be excluded, and further research is required in future work to empirically demonstrate or disprove this.

Moreover, aberrant expression of CTNNB1, which is the epigenetically modified protein, induces malignant pathways in normal cells and abnormal activity of CTNNB1 also exists in malignant progression [57]. In stem cells, expression of CTNNB1 might serve as a multifunctional protein with a central role in stem cell renewal and differentiation [58], [59]. Recent studies have shown that CTNNB1 could regulate Tert expression through the interaction with Klf4, and thereby telomere length, which could be critical in human cancer [60]. Here, enhanced expression of CTNNB1 and increased expression target genes accompanied with more serious transformation in hESCs implied its role in stem cell-derived tumor initiation and progression (Figure 6 and Figure 10). Clearly, further work is needed to fully appreciate its function on malignant transformation of stem cells. As for VIM, it is one of the mammalian intermediate filament proteins and a feature of proliferating fetal cells. VIM expression increased in karyotypically abnormal hESCs showed an increased protein level along with increased gene expression levels (Figure 9) even in optimal culture condition. This suggested that vimentin might be relevant for cellular adaptation to culture conditions and reflects the progressive adaptation of self-renewing cells to their culture conditions.

## Conclusions

We have shown for the first time the use of ITRAQ-based tandem mass spectrometry to quantify proteins of normal and aberrant karyotypic hESCs from simple to more complex karyotype abnormalities, the purpose of which was to elucidate the dynamics of the malignant transformation process seen in hESCs. This study should serve as a useful resource for the discovery of transformed phenotypic biomarkers for further monitoring the safety of the clinical use of hESCs. Increased expression of HDAC2 and CTNNB1 might serve as potential prognostic markers in the malignant transformation of hESCs and could be detected as early as the pre-neoplastic stage.

## Supporting Information

Figure S1 **G-banding and characterization of** ***ch*** **HES-3 cells at different karyotypic stages.** (A) showing normal karyotype of 46, XX at passage 30. (B) showing abnormal karyotype of 46, XX, dup (1) (p32p36) at passage 72. (C) showing abnormal karyotype with complex chromosomal rearrangement involving chromosomes 1, 2, 4, 6, 7, 8, 10 and chromosome 15 at passage 182. This complex rearrangement contains a reciprocal translocation between chromosome 1, 6 and 4, and as well as an insertion segment of 1q21q25 into the band 4p16; a derivative chromosome 2 resulted from two reciprocal translocations, one is positioned between chromosomes 2 and 7 and the other is positioned between the same chromosome 7 and chromosome 8; a inversion of chromosome 10, and a derivative chromosome 15 resulted from a reciprocal translocation between chromosomes 4 and 15. This is seen as 46,XX,dup(1)(p32p36)t(1;6;4)(q25;q23;p16)ins(4;1)(p16;q21q25), der(2)t(2;7)(q35;qter)t(7;8)(q22; q22),inv(10)(p11q21),der(15)t(4;15)(q21;q26). (D–F) shows identical morphology of normal *ch*HES-3 (Normal) cells, simple duplication *ch*HES-3 (SIMP) cells, and karyotypically complex *ch*HES-3 (COMP) colonies. (G–I) shows undifferentiated hES cells that stained positively for alkaline phosphatase activity. (J-AA) shows all colonies staining positive for the following specific molecular markers: OCT4 (J–L), TRA-1-60 (M–O), TRA-1-81 (P–R), SSEA-4 (S–U) and SSEA-3 (V–X). By contrast colonies stained negative for SSEA-1 (Y-AA). All images were captured at ×100 magnification.
(TIF)

Figure S2 **Gray scale ratio of differentially expressed proteins in six pairs of hESCs.** Gray scale ratio of differentially expressed proteins in six pairs of hESC lines of early and late passages by Western blot analysis. The straps of all Western blot data were applied in the analysis of the data by using the Image J system to calculate their gray-scale ratio relative to β-ACTIN. Data are represented as mean ± S.D. (n = 3). The values *P<0.05, and **P<0.01 are described with respect to the protein expression in early passages of hESC lines.
(TIF)

Figure S3 **Germ layers markers detection of hESC lines at different passages of differentiation** ***in vitro*** **.** Real-time quantitative RT-PCR analysis of the germ layers markers in day 21 embryoid bodies. The expression of eight marker genes representing different germ layers including ectoderm genes *KRT17* (Keratin 17) and *PAX6* (Paired box 6), mesoderm genes *RUNX1* (runt-related transcription factor 1) and *HAND1* (heart and neural crest derivatives expressed 1), endoderm genes *AFP* (alpha-fetoprotein) and *SOX17* (SRY (sex determining region Y)-box 17), and trophoblast marker genes *CDX2* (caudal type homeobox 2) and *CGB5* (Chorionic Gonadotropin, Beta Polypeptide 5) were compared in day-21 embryoid bodies of early and late passages hESCs (*ch*HES-8 P11/P34, *ch*HES-22 P35/P98, *ch*HES-69 P13/P27). Data are represented as mean ± S.D. (n = 3). *P<0.05, **P<0.01 with respect to the mRNA expression in three early passages of hESC lines by independent sample t tests.
(TIF)

Table S1 **Antibodies used for immunocytochemical staining and Western blot analysis.**
(DOC)

Table S2 **Primers Used for Real-Time RT-PCR.**
(DOC)

Table S3 **Primers Used for Real-Time PCR.**
(DOC)

Table S4 **A total of 2583 proteins were detected with 95% confidence.**
(XLS)

Table S5 **A total of 316 differentially expressed proteins.**
(XLS)

Table S6 **Microarray probe set data of 2583 proteins as detected by iTRAQ analysis.**
(XLS)
