# Supplementary figures and images for: Identification of Proteins Related to Epigenetic Regulation in the Malignant Transformation of Aberrant Karyotypic Human Embryonic Stem Cells by Quantitative Proteomics
Source: PLoS One. 2014 Jan 17;9(1):e85823. doi: 10.1371/journal.pone.0085823 (PMC3895013; doi:10.1371/journal.pone.0085823)

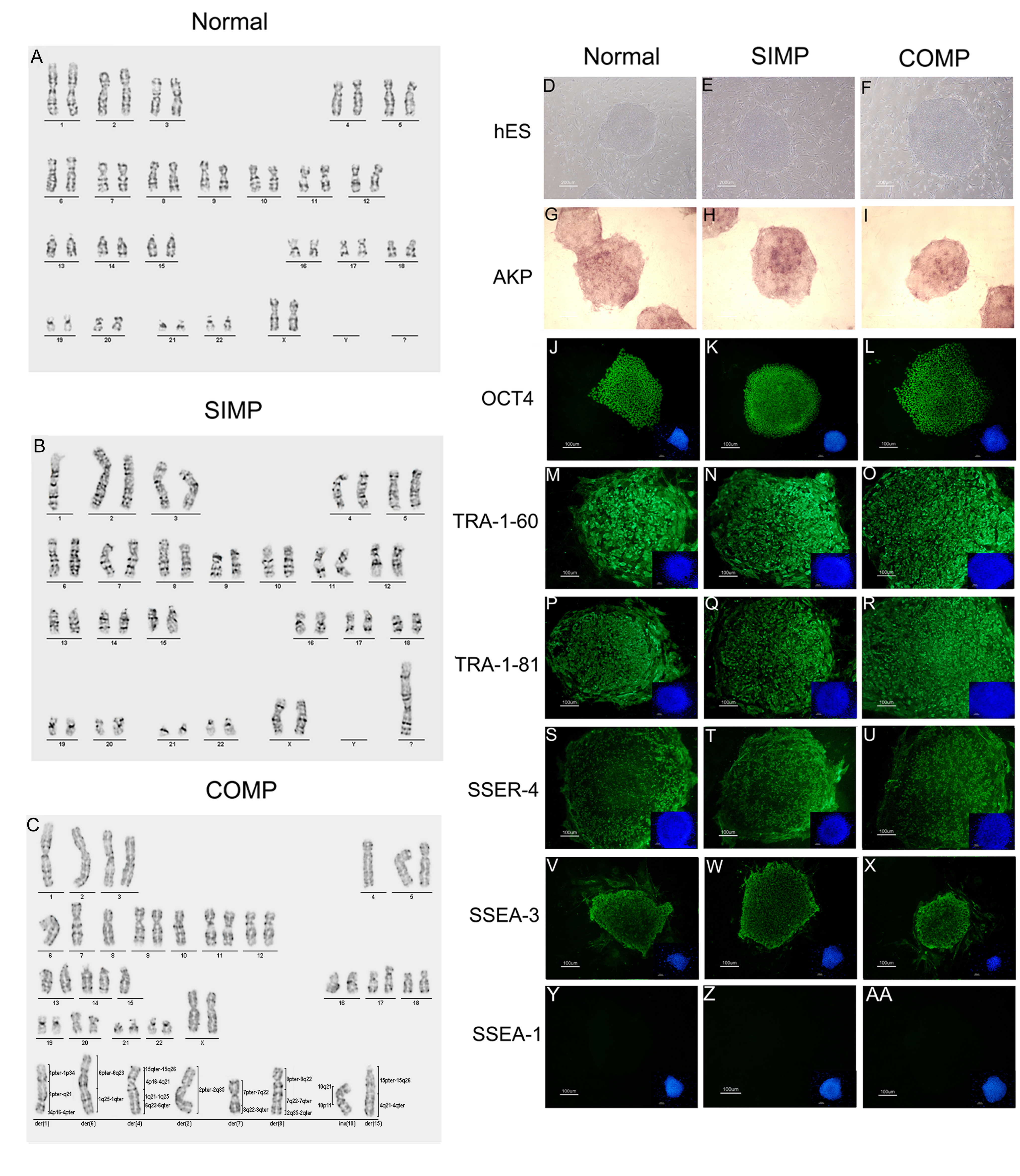

Supplement: Figure S1 — G-banding and characterization of ch HES-3 cells at different karyotypic stages. (A) showing normal karyotype of 46, XX at passage 30. (B) showing abnormal karyotype of 46, XX, dup (1) (p32p36) at passage 72. (C) showing abnormal karyotype with complex chromosomal rearrangement involving chromosomes 1, 2, 4, 6, 7, 8, 10 and chromosome 15 at passage 182. This complex rearrangement contains a reciprocal translocation between chromosome 1, 6 and 4, and as well as an insertion segment of 1q21q25 into the band 4p16; a derivative chromosome 2 resulted from two reciprocal translocations, one is positioned between chromosomes 2 and 7 and the other is positioned between the same chromosome 7 and chromosome 8; a inversion of chromosome 10, and a derivative chromosome 15 resulted from a reciprocal translocation between chromosomes 4 and 15. This is seen as 46,XX,dup(1)(p32p36)t(1;6;4)(q25;q23;p16)ins(4;1)(p16;q21q25), der(2)t(2;7)(q35;qter)t(7;8)(q22; q22),inv(10)(p11q21),der(15)t(4;15)(q21;q26). (D–F) shows identical morphology of normal chHES-3 (Normal) cells, simple duplication chHES-3 (SIMP) cells, and karyotypically complex chHES-3 (COMP) colonies. (G–I) shows undifferentiated hES cells that stained positively for alkaline phosphatase activity. (J-AA) shows all colonies staining positive for the following specific molecular markers: OCT4 (J–L), TRA-1-60 (M–O), TRA-1-81 (P–R), SSEA-4 (S–U) and SSEA-3 (V–X). By contrast colonies stained negative for SSEA-1 (Y-AA). All images were captured at ×100 magnification. (TIF) [file pone.0085823.s001.tif]

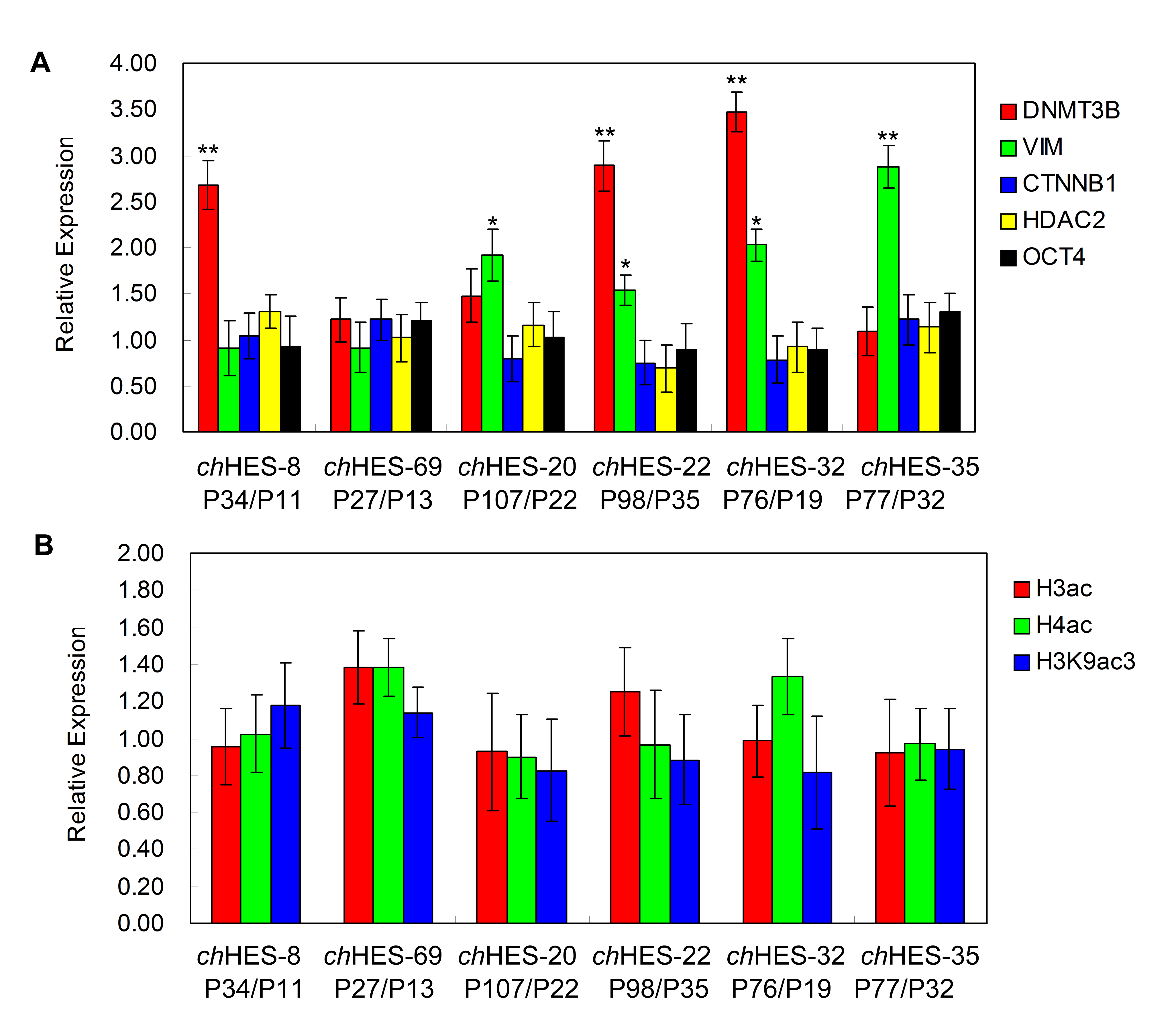

Supplement: Figure S2 — Gray scale ratio of differentially expressed proteins in six pairs of hESCs. Gray scale ratio of differentially expressed proteins in six pairs of hESC lines of early and late passages by Western blot analysis. The straps of all Western blot data were applied in the analysis of the data by using the Image J system to calculate their gray-scale ratio relative to β-ACTIN. Data are represented as mean ± S.D. (n = 3). The values *P<0.05, and **P<0.01 are described with respect to the protein expression in early passages of hESC lines. (TIF) [file pone.0085823.s002.tif]
