# Supplementary material for: Identification of Proteins Related to Epigenetic Regulation in the Malignant Transformation of Aberrant Karyotypic Human Embryonic Stem Cells by Quantitative Proteomics
Source: PLoS One. 2014 Jan 17;9(1):e85823. doi: 10.1371/journal.pone.0085823 (PMC3895013; doi:10.1371/journal.pone.0085823)
Supplement: Table S1 — Antibodies used for immunocytochemical staining and Western blot analysis. (DOC) [file pone.0085823.s004.doc]

**Table S1. Primary antibodies used for immunocytochemical staining and western blot analysis** of human ESCs

| **Protein** | **Host** | **Vendor** |
| --- | --- | --- |
| OCT4 | Mouse monoclonal | Santa Cruz Biotechnology |
| TRA-1-60 | Mouse monoclonal | Chemicon |
| TRA-1-81 | Mouse monoclonal | Chemicon |
| SSEA-3 | Rat monoclonal | Santa Cruz Biotechnology |
| SSEA-4 | Mouse monoclonal | R&D Systems |
| SSEA-1 | Mouse monoclonal | R&D Systems |
| DNMT3B | Rabbit polyclonal | Abcam |
| DNMT3A | Rabbit polyclonal | Cell signaling Technology |
| CTNNB1 | Rabbit monoclonal | Santa Cruz Biotechnology |
| HDAC2 | Rabbit monoclonal | Santa Cruz Biotechnology |
| VIM | Mouse monoclonal | Santa Cruz Biotechnology |
| NES | Rabbit polyclonal | Abcam |
| HSPA1A | Mouse monoclonal | Santa Cruz Biotechnology |
| HIST1H1B | Rabbit monoclonal | Abcam |
| H3K9ac3 | Rabbit polyclonal | Cell signaling Technology |
| H3ac | Rabbit polyclonal | Millipore |
| H4ac | Rabbit polyclonal | Millipore |
| H4K12ac | H4K12ac | Millipore |
| β-Actin | Mouse monoclonal | Sigma |
