# Supplementary material for: Identification of Proteins Related to Epigenetic Regulation in the Malignant Transformation of Aberrant Karyotypic Human Embryonic Stem Cells by Quantitative Proteomics
Source: PLoS One. 2014 Jan 17;9(1):e85823. doi: 10.1371/journal.pone.0085823 (PMC3895013; doi:10.1371/journal.pone.0085823)
Supplement: Table S2 — Primers Used for Real-Time RT-PCR. (DOC) [file pone.0085823.s005.doc]

**Table S2. Primers Used for** Real-Time RT-PCR

| *RAP2B* | **Forward Sequence:** | CGCAAGGAGATTGAGGTGGACT |
| --- | --- | --- |
|  | **Reverse Sequence:** | TTGACGAGGCTGTAGACCAGGA |
| *RAB1A* | **Forward Sequence:** | GGGAACAAATGTGATCTGACCAC |
|  | **Reverse Sequence:** | GAAAGACTGTTCTACATTCGTTGC |
| *PRDX1* | **Forward Sequence:** | CTGCCAAGTGATTGGTGCTTCTG |
|  | **Reverse Sequence:** | AATGGTGCGCTTCGGGTCTGAT |
| *CSNK2A1* | **Forward Sequence:** | GGTGAGGATAGCCAAGGTTCTG |
|  | **Reverse Sequence:** | TCACTGTGGACAAAGCGTTCCC |
| *KPNB1* | **Forward Sequence:** | CTGCTTCCTGAAGCTGCCATCA |
|  | **Reverse Sequence:** | CTTCAGCCAGACTGGAGAAAGC |
| *VIM* | **Forward Sequence:** | AGGCAAAGCAGGAGTCCACTGA |
|  | **Reverse Sequence:** | ATCTGGCGTTCCAGGGACTCAT |
| *LMNA* | **Forward Sequence:** | ATGAGGACCAGGTGGAGCAGTA |
|  | **Reverse Sequence:** | ACCAGGTTGCTGTTCCTCTCAG |
| *VDAC2* | **Forward Sequence:** | CTTTGCAGTGGGCTACAGGACT |
|  | **Reverse Sequence:** | CGAGTGCAGTTGGTACCTGATG |
| *ADSS* | **Forward Sequence:** | GTGCCTTTCCTACAGAGCAAGAC |
|  | **Reverse Sequence:** | AAACGAGGTCCAACCAGCCACA |
| *YWHAE* | **Forward Sequence:** | GACAGAACTTCCACCAACGCATC |
|  | **Reverse Sequence:** | CACTCAGCGTATCCAGTTCTGC |
| *BGN* | **Forward Sequence:** | TTGAACCTGGAGCCTTCGATGG |
|  | **Reverse Sequence:** | TTGGAGTAGCGAAGCAGGTCCT |
| *API5* | **Forward Sequence:** | GAACAGACCTTCAATCCCTCGG |
|  | **Reverse Sequence:** | GGTTAGGGAGAACCTGCTCACA |
| *THY1* | **Forward Sequence:** | GAAGGTCCTCTACTTATCCGCC |
|  | **Reverse Sequence:** | TGATGCCCTCACACTTGACCAG |
| *HMGB1* | **Forward Sequence:** | GCGAAGAAACTGGGAGAGATGTG |
|  | **Reverse Sequence:** | GCATCAGGCTTTCCTTTAGCTCG |
| *SUB1* | **Forward Sequence:** | AATGAGGTACGTTAGTGTTCGCG |
|  | **Reverse Sequence:** | CTTCAGCTGGCTCCATTGTTCTG |
| *PSIP1* | **Forward Sequence:** | AGGCAGGAGTAGTGACAACAGC |
|  | **Reverse Sequence:** | CTCTCTGAAGGACAGGGCTGTT |
| *CARHSP1* | **Forward Sequence:** | CCCGTCTACAAAGGAGTCTGCA |
|  | **Reverse Sequence:** | TATAGGTGACCTCGTCGCCTTC |
| *DNMT1* | **Forward Sequence:** | AGGTGGAGAGTTATGACGAGGC |
|  | **Reverse Sequence:** | GGTAGAATGCCTGATGGTCTGC |
| *EIF3D* | **Forward Sequence:** | CGCTACTTGGAAGTATCAGAGCC |
|  | **Reverse Sequence:** | TGGTGACAGTGTGGAAGATGCG |
| *HIST1H1B* | **Forward Sequence:** | CCGAAAAAGGCAACCAAGAGTCC |
|  | **Reverse Sequence:** | GTTTTCACACGCCAGCTTCCTAC |
| *HNRNPD* | **Forward Sequence:** | GCCAAGGTTACGGTGGTTATGG |
|  | **Reverse Sequence:** | TGATGACCACCTCGCCTGGATA |
| *DNMT3B* | **Forward Sequence:** | TAACAACGGCAAAGACCGAGGG |
|  | **Reverse Sequence:** | TCCTGCCACAAGACAAACAGCC |
| *DNMT3A* | **Forward Sequence:** | CCTCTTCGTTGGAGGAATGTGC |
|  | **Reverse Sequence:** | GTTTCCGCACATGAGCACCTCA |
| *CTNNB1* | **Forward Sequence:** | CACAAGCAGAGTGCTGAAGGTG |
|  | **Reverse Sequence:** | GATTCCTGAGAGTCCAAAGACAG |
| *HDAC2* | **Forward Sequence:** | CTCATGCACCTGGTGTCCAGAT |
|  | **Reverse Sequence:** | GCTATCCGCTTGTCTGATGCTC |
| *SMAD2* | **Forward Sequence:** | GGGTTTTGAAGCCGTCTATCAGC |
|  | **Reverse Sequence:** | CCAACCACTGTAGAGGTCCATTC |
| *NES* | **Forward Sequence:** | TCAAGATGTCCCTCAGCCTGGA |
|  | **Reverse Sequence:** | AAGCTGAGGGAAGTCTTGGAGC |
| *HSPA1A* | **Forward Sequence:** | ACCTTCGACGTGTCCATCCTGA |
|  | **Reverse Sequence:** | TCCTCCACGAAGTGGTTCACCA |
| *28S* | **Forward Sequence:** | GAACTTTGAAGGCCGAAGTG |
|  | **Reverse Sequence:** | ATCTGAACCCGACTCCCTTT |
| *KRT17* | **Forward Sequence:** | GGTGGGTGGTGAGATCAATGT |
|  | **Reverse Sequence:** | CGCGGTTCAGTTCCTCTGTC |
| *PAX6* | **Forward Sequence:** | AACAGACACAGCCCTCACAAACA |
|  | **Reverse Sequence:** | CGGGAACTTGAACTGGAACTGAC |
| *RUNX1* | **Forward Sequence:** | CTGCCCATCGCTTTCAAGGT |
|  | **Reverse Sequence:** | GCCGAGTAGTTTTCATCATTGCC |
| *HAND1* | **Forward Sequence:** | AAGAGAACCAGACGCAGGAA |
|  | **Reverse Sequence:** | GGCAGGATGAACAAACACCT |
| *AFP* | **Forward Sequence:** | CCATGTACATGAGCACTGTTG |
|  | **Reverse Sequence:** | CTCCAATAACTCCTGGTATCC |
| *SOX17* | **Forward Sequence:** | CAGTGACGACCAGAGCCAGACC |
|  | **Reverse Sequence:** | CCACGACTTGCCCAGCATCTT |
| *CDX2* | **Forward Sequence:** | CCGCAGAGCAAAGGAGA |
|  | **Reverse Sequence:** | CAGGGACAGAGCCAGACA |
| *CGB5* | **Forward Sequence:** | GTCAACACCACCATCTGTGC |
|  | **Reverse Sequence:** | GGCCTTTGAGGAAGAGGAGT |
| *OCT4* | **Forward Sequence:** | AGCGAACCAGTATCGAGAAC |
|  | **Reverse Sequence:** | TTACAGAACCACACTCGGAC |
| *NANOG* | **Forward Sequence:** | TGAACCTCAGCTACAAACAG |
|  | **Reverse Sequence:** | TGGTGGTAGGAAGAGTAAAG |
| *CCND1* | **Forward Sequence:** | TCTACACCGACAACTCCATCCG |
|  | **Reverse Sequence:** | TCTGGCATTTTGGAGAGGAAGTG |
| *MYC* | **Forward Sequence:** | CCTGGTGCTCCATGAGGAGAC |
|  | **Reverse Sequence:** | CAGACTCTGACCTTTTGCCAGG |

FOOTNOTE: These real-time primers were refer to OriGene (<http://www.origene.com/>)
