# Supplementary material for: Identification of Proteins Related to Epigenetic Regulation in the Malignant Transformation of Aberrant Karyotypic Human Embryonic Stem Cells by Quantitative Proteomics
Source: PLoS One. 2014 Jan 17;9(1):e85823. doi: 10.1371/journal.pone.0085823 (PMC3895013; doi:10.1371/journal.pone.0085823)
Supplement: Table S3 — Primers Used for Real-Time PCR. (DOC) [file pone.0085823.s006.doc]

**Table S3. Primers Used for gene copy number analysis**

| *CTNNB1* | **Forward Sequence:** | TCTCCTCAGATGGTGTCTGCT |
| --- | --- | --- |
|  | **Reverse Sequence:** | TTACCCAAGCATTTTCACCAG |
| *DNMT3B* | **Forward Sequence:** | GCTGTTTGTCTTGTGGCAGG |
|  | **Reverse Sequence:** | GAGGGGAGTTAGAGGAGGCA |
| *HDAC2* | **Forward Sequence:** | CAAATGTCGGTCCCTCCTCC |
|  | **Reverse Sequence:** | CCATGGCGTACAGTCAAGGA |
| *VIM* | **Forward Sequence:** | ACGTCTTGACCTTGAACGCA |
|  | **Reverse Sequence:** | GGCTGCCTTACCCTCATTCA |
| *β-Globin* | **Forward Sequence:** | AAAGGTGCCCTTGAGGTTGTC |
|  | **Reverse Sequence:** | TGAAGGCTCATGGCAAGAAA |
